# Harnessing the power of diffusion models for plant disease image augmentation

**DOI:** 10.3389/fpls.2023.1280496

**Published:** 2023-11-07

**Authors:** Abdullah Muhammad, Zafar Salman, Kiseong Lee, Dongil Han

**Affiliations:** Vision and Image Processing Lab, Department of Computer Engineering, Sejong University, Seoul, Republic of Korea

**Keywords:** plant science, plant disease, data augmentation, generative AI, GAN, diffusion, vision transformers, leaf segmentation

## Abstract

**Introduction:**

The challenges associated with data availability, class imbalance, and the need for data augmentation are well-recognized in the field of plant disease detection. The collection of large-scale datasets for plant diseases is particularly demanding due to seasonal and geographical constraints, leading to significant cost and time investments. Traditional data augmentation techniques, such as cropping, resizing, and rotation, have been largely supplanted by more advanced methods. In particular, the utilization of Generative Adversarial Networks (GANs) for the creation of realistic synthetic images has become a focal point of contemporary research, addressing issues related to data scarcity and class imbalance in the training of deep learning models. Recently, the emergence of diffusion models has captivated the scientific community, offering superior and realistic output compared to GANs. Despite these advancements, the application of diffusion models in the domain of plant science remains an unexplored frontier, presenting an opportunity for groundbreaking contributions.

**Methods:**

In this study, we delve into the principles of diffusion technology, contrasting its methodology and performance with state-of-the-art GAN solutions, specifically examining the guided inference model of GANs, named InstaGAN, and a diffusion-based model, RePaint. Both models utilize segmentation masks to guide the generation process, albeit with distinct principles. For a fair comparison, a subset of the PlantVillage dataset is used, containing two disease classes of tomato leaves and three disease classes of grape leaf diseases, as results on these classes have been published in other publications.

**Results:**

Quantitatively, RePaint demonstrated superior performance over InstaGAN, with average Fréchet Inception Distance (FID) score of 138.28 and Kernel Inception Distance (KID) score of 0.089 ± (0.002), compared to InstaGAN’s average FID and KID scores of 206.02 and 0.159 ± (0.004) respectively. Additionally, RePaint’s FID scores for grape leaf diseases were 69.05, outperforming other published methods such as DCGAN (309.376), LeafGAN (178.256), and InstaGAN (114.28). For tomato leaf diseases, RePaint achieved an FID score of 161.35, surpassing other methods like WGAN (226.08), SAGAN (229.7233), and InstaGAN (236.61).

**Discussion:**

This study offers valuable insights into the potential of diffusion models for data augmentation in plant disease detection, paving the way for future research in this promising field.

## 1 Introduction

The advent of artificial intelligence (AI) has revolutionized numerous fields, including plant sciences. AI’s potential to automate and optimize various tasks has been harnessed to address some of the most pressing challenges in plant sciences, such as disease detection and classification [Ahmad et al. (2018)]. The historical progression of AI in plant sciences can be traced back to the early applications of machine learning algorithms for tasks such as plant classification and disease detection. These initial applications primarily relied on handcrafted features extracted from plant images, which were then used to train machine learning models.

The emergence of computer vision technologies marked a significant milestone in the use of AI in plant sciences. Computer vision, a field that enables computers to gain a high-level understanding from digital images or videos, has been instrumental in automating the process of disease detection and classification in plants. The application of computer vision in plant sciences has been facilitated by the development of Convolutional Neural Networks (CNNs), which have shown remarkable success in image classification tasks [Salman et al. (2023)]. The successful application of computer vision technologies in plant sciences is heavily reliant on the existence of broad and diverse datasets. Compared to regular computer vision tasks, amassing a large amount of plant disease image data can be a daunting task. Labeling plant disease data needs a good understanding of biology. Also, to get top-quality disease data, plants have to be grown in a very controlled and separate area to keep them from getting contaminated. This process involves a lot of work, costs and restrictions due to seasonal changes and geographical locations. Datasets for plant diseases are often uneven, and things like weather, temperature, and bugs that carry diseases can greatly affect how diseases develop. Some diseases are hard to gather data on, and the data that is collected often has uneven amounts for each class of disease. Such datasets often exhibit a skew in representation, with some disease classes being over-represented [Ahmad et al. (2021)]. To mitigate these issues, the concept of data augmentation has been introduced. Data augmentation strategies enhance datasets by creating varied versions of the original images, using methods such as cropping, resizing, and rotation. This not only boosts the quantity of available training data but also introduces an element of diversity. This diversity aids in improving the model’s ability to generalize, thereby enhancing its performance on unseen data.

The image features that give clues for diagnosis are often much smaller than in general object recognition problems. For example, in the early stages of a disease, the only signs might be just a tiny dot or faint lines in the image. Diagnosing plants based on images is very hard because it requires recognizing very fine details. Usually, a deep learning model like a CNN looks at the big picture of an image, like its brightness or color, rather than small details that might show a disease. Also, when testing a model using different sets of data (training, validation, and test sets), things like the background or brightness of the images can make the model seem more accurate than it really is. This might result in another form of overfitting, such that it works well in one situation but not in others. For example, a model might be 86% accurate at diagnosing a disease in cucumbers on one farm but only 20.7% accurate on a different farm.

Generally, there isn’t a lot of variety in pictures of diseased plants, especially if they’re grown in a controlled environment. But it’s usually easy to get pictures of healthy plants. So, we think that if we can turn pictures of healthy plants into pictures of diseased plants, we can create a more varied and reliable dataset. This could make diagnosing diseases more accurate and also make it cheaper to label the data. Image Inpainting techniques, which have seen significant advancements in recent years, offer the potential to fill this gap. By applying these techniques, it is possible to create realistic simulations of plant diseases on healthy leaves, thereby enriching the dataset and enhancing the model’s ability to generalize across different scenarios.

Image Inpainting, sometimes called Image Completion, is like filling in a puzzle where pieces are missing. It’s about adding parts to an image so that everything fits together perfectly and looks natural. One of the most effective and widely used tools for this job is called Generative Adversarial Networks (GANs), introduced by Goodfellow et al. (2014). GANs are a class of artificial intelligence algorithms that use two neural networks, a generator, and a discriminator, contesting with each other in a zero-sum game framework. They are capable of generating synthetic images that are almost indistinguishable from real images, providing a powerful tool for data augmentation. Imagine having a brush that knows exactly how to paint flowers, leaves, or faces. Some methods make sure that the filled-in parts don’t all look the same. This is important because we don’t want every leaf or tree to look identical. Some new techniques are being developed to make sure there’s a good balance between making things look real and adding some variety. Some variations of GANs like StyleGAN by Karras et al. (2019) and CycleGAN by Zhu et al. (2017) gained huge popularity due to their superior results, especially in style transfer, which is another useful technique in image processing. Consider discoloration or patterns on a leaf as a style template for a particular disease. In such cases, this ability to perform style transfer can be used to create artificial disease symptoms in healthy leaf images in order to fill the gap between under-represented and over-represented classes. However, these methods may result in unwanted artifacts in unwanted locations, such as disease symptoms on the ground or any other object in the background. Therefore, in this research, we performed experiments on an instance-aware generative adversarial network, InstaGAN by Mo et al. (2019). This method uses instance segmentation masks to guide the creation of images but does not directly use them for filling in missing parts.

Diffusion models have emerged as a prominent approach in the field of AI, specifically in image generation, and have become a notable rival to Generative Adversarial Networks (GANs). RePaint by Lugmayr et al. (2022) is a cutting-edge approach to free-form inpainting that is built upon Denoising Diffusion Implicit Models (DDIM) by Song et al. (2021). The structure of DDIM consists of two main components: a forward diffusion process and a reverse diffusion process. In the forward diffusion process, the original data is gradually corrupted by adding noise at each step, following a carefully designed noise schedule. This process transforms the data into pure noise over a series of timesteps. In the reverse diffusion process, the model learns to reverse this transformation, starting from noise and gradually denoising it to generate new samples that resemble the original data. RePaint starts with the original image and applies a forward diffusion process, corrupting the specified regions (masks) with noise. In the reverse process, RePaint utilizes a pretrained unconditional DDIM as the generative prior. By altering only the reverse diffusion iterations, it reconstructs the image, filling in the masked regions with new content that blends seamlessly with the surrounding areas.

The techniques described above can be applied to the creation of disease images from healthy leaf images. By utilizing advanced inpainting methods, it is possible to simulate the appearance of plant diseases on healthy leaves. This can be particularly useful in building diverse and reliable disease datasets for plant diagnosis. The ability to transform healthy images into disease cases can improve the performance of diagnosis models and reduce the cost of labeling, contributing to more effective and efficient plant disease management. In light of the evolving landscape of image generation and the transition from traditional GANs to diffusion models, this paper makes several key contributions to the field. These insights not only deepen our understanding of the underlying principles of models like InstaGAN and RePaint but also demonstrate their practical applications in areas such as plant disease detection. The specific contributions of this study are as follows:

- **Comparative Analysis**: Provides a detailed comparison of diffusion models, specifically DDIM and RePaint, with GAN-based methods, including InstaGAN, in the context of plant disease image augmentation.
- **Application to Agriculture**: Demonstrates the application of these models to plant disease detection, using a subset of the PlantVillage dataset for a fair and relevant evaluation.
- **Quantitative Evaluation**: Introduces quantitative measures such as FID [Heusel et al. (2017)], KID [Binkowski et al. (2018)], IS [Salimans et al. (2016)], PSNR [Ledig et al. (2017)], and SSIM [Wang et al. (2004)] for an objective assessment of model performance.
- **In-Depth Exploration**: Offers an in-depth exploration of InstaGAN and RePaint, including their underlying principles, structures, and advantages.
- **Contribution to Literature**: Highlights the chronological development of diffusion models, contributing valuable insights into the field of AI and image generation.
- **Practical Implications**: Emphasizes the practical implications of the findings, with potential applications in various industries including agriculture and healthcare.

These contributions collectively enhance our understanding of diffusion models and their application in image generation and augmentation, offering valuable insights for both academic research and practical implementation.

## 2 Background

### 2.1 Generative adversarial networks

The advent of Generative Adversarial Networks (GANs) marked a significant advancement in generative AI technology. The Generator’s goal is to create data that is indistinguishable from real data. It takes random noise as input and generates samples as output. The aim is to improve its ability to create fake data by learning from the Discriminator’s feedback. The Discriminator’s goal is to distinguish between real data from the training set and fake data created by the Generator. It takes in both real and fake samples and assigns a probability that a given sample is real. During training, the Generator and Discriminator are in a continuous game where the Generator tries to produce fake data that looks as real as possible, and the Discriminator tries to get better at distinguishing real data from fake. This process leads to the Generator creating highly realistic data. The primary purpose of GANs extends beyond merely creating realistic fake data; it leverages this capability for various practical applications that can benefit different fields and industries. These applications encompass a wide range of tasks, including the creation of realistic images such as faces that do not exist, data augmentation (particularly valuable when limited real data is available), transferring the style of one image to another (such as converting a photo into a painting), enhancing the resolution of images (known as Super-Resolution), generating molecular structures for potential new drugs (a key component in Drug Discovery), and creating realistic voice recordings.

While the original GANs provided a novel way to generate data, they suffered from training instability and mode collapse. To address these limitations, Conditional Generative Adversarial Nets (cGANs) were introduced by Mirza and Osindero (2014), allowing the model to generate data conditioned on certain information, thereby making the data generation process more controlled. This approach mitigated some of the training issues but still faced challenges in generating complex data structures. The introduction of Deep Convolutional Generative Adversarial Networks (DCGANs) by Radford et al. (2015) further advanced the field by utilizing convolutional layers in both the generator and discriminator, making them more suitable for image generation. Wasserstein GAN (WGAN) by Arjovsky et al. (2017) introduced a different loss function that provided more stable training and helped to solve the vanishing gradient problem, a significant improvement over previous methods. Cycle-Consistent Adversarial Networks (CycleGAN) by Zhu et al. (2017) enabled image-to-image translation without paired examples, such as applying facial disguise i.e. glasses, mask, and beard on another person’s face, addressing the limitation of needing paired training data in previous models (Ahmad et al. (2022)). However, CycleGANs could suffer from artifacts in the translated images. Recent advancements such as BigGAN by Brock et al. (2018) have focused on generating high-fidelity and diverse images at a large scale, pushing the boundaries of what GANs can achieve. The field continues to evolve with innovations like StyleGAN by Karras et al. (2019), a style control on the generated images, allowing for fine-grained control over the appearance of the generated data. While StyleGAN provides unprecedented control, it also introduces new challenges in understanding and manipulating the latent space. StarGAN by Choi et al. (2017) introduced a novel and scalable approach that uses a single model to perform image-to-image translations for multiple domains.

Combining the concepts of cGANs and CycleGAN, Mo et al. (2019) introduced InstaGAN, which incorporates instance-level information into image-to-image translation through the use of instance segmentation masks, allowing for more precise control over individual objects within the scene. These masks enable InstaGAN to selectively target specific regions of the image, enhancing the translation accuracy and flexibility. InstaGAN’s approach of building upon the CycleGAN framework and drawing inspiration from conditional GANs represents a novel and powerful combination. By leveraging the global transformation capabilities of CycleGAN and the targeted control offered by cGANs, InstaGAN introduces a more nuanced and flexible approach to image-to-image translation. This enables a wide range of creative and practical applications, from object transfiguration to style transfer, and represents a significant contribution to the field of generative models.

### 2.2 Diffusion models

Diffusion models, also known as score-based generative models, are rooted in the idea of modeling the data distribution directly using a noise process. In the context of AI and image generation, diffusion models have been explored as a way to create realistic and high-quality images by modeling the data distribution directly. This approach contrasts with GANs, which rely on a generator-discriminator framework to create synthetic data. Diffusion models have become a rival to GANs due to several key factors. GANs are known for their training instability, where small changes in hyperparameters can lead to vastly different results. Diffusion models, on the other hand, have shown more stable training behavior. Diffusion models have demonstrated the ability to generate high-quality images that rival or even surpass those produced by state-of-the-art GANs. Diffusion models also offer flexibility in modeling different data distributions, making them applicable to a wide range of tasks beyond image generation.

Diffusion models often have a simpler architecture and training process compared to GANs, which require careful balancing between the generator and discriminator. Diffusion models tend to be more robust to hyperparameter choices and are less prone to common GAN issues such as mode collapse. The diffusion process provides a clear and interpretable way to understand how data is generated, unlike the more opaque process of GANs. Some studies have shown that diffusion models may generalize better to unseen data, making them a valuable tool for tasks such as data augmentation. The paper “High-Resolution Image Synthesis with Latent Diffusion Models” by Rombach et al. (2022) marked a significant step forward by achieving state-of-the-art synthesis results on image data through diffusion models (DMs). This approach greatly boosted visual fidelity. Following this, Choi et al. (2021) introduced Iterative Latent Variable Refinement (ILVR), guiding the generative process in Denoising Diffusion Probabilistic Models (DDPM) [Ho et al. (2020)] to generate high-quality images based on a given reference image. This method enabled a single DDPM to sample images from various sets. Various studies have proven that DMs offers a more stable training process compared to traditional GANs [Muhammad et al. (2023)]. The diffusion process provides a clear and smooth path from the data to noise, making the learning of the reverse process more tractable [Dhariwal and Nichol (2021)].

RePaint by Lugmayr et al. (2022) takes image inpainting to a new level. RePaint leverages the structure and principles of DDPM to achieve high-quality inpainting. Arbitrary binary masks are used to specify the regions for inpainting. The forward and reverse diffusion processes of DDPM are used to model the data distribution and generate new content within specified regions of an image. RePaint offers fine-grained control over the inpainting process, allowing for targeted modifications within specific regions defined by the masks. Unlike traditional methods that train for specific mask distributions, RePaint can handle even extreme masks, providing flexibility in the inpainting process. By employing a pretrained unconditional DDPM, RePaint doesn’t require paired examples for training. This allows it to generate diverse and high-quality output images for any inpainting form.

## 3 Related work

GANs have been used to generate synthetic images of plant diseases, addressing the issue of class imbalance and enhancing the robustness of disease detection models. Several studies have proposed modifications and improvements in the original GAN architecture to address limitations of GANs such as mode collapse, training instability and other issues faced in plant disease data modeling.

Bi and Hu (2020) utilized improved the training stability of Wasserstein GANs for complex image generation such plant disease images. LR-GAN by Yang et al. (2016) further extended GANs with LRGAN, introducing layered recursive networks for image generation. The introduction of Self-attention Generative Adversarial Networks (SAGAN) by Zhang et al. (2018) marked a significant advancement by enhancing the focus on specific regions of images. A substantial leap towards plant-specific image synthesis was made with the work on Two Pathway Encoder GAN Yilma et al. (2020), providing a novel architecture focusing on data generation. Zhang et al. (2022) introduced MMDGAN, a fusion data augmentation method for tomato-leaf disease identification. Abbas et al. (2021) further refined this approach by utilizing transfer learning with C-GAN for tomato plant disease detection. In 2022, the introduction of LeafGAN by Cap et al. (2022) marked a turning point by providing a versatile and effective tool specifically designed for plant disease image augmentation. The most recent advancements include hybrid approaches such as the combination of E-GAN and CapsNet by Vasudevan and Karthick (2023), PiiGAN by for pluralistic image inpainting, and Fine Grained-GAN for grape leaf spot identification by Zhou et al. (2021). These methods collectively enhanced the robustness, diversity, and realism of image generation, significantly advancing data augmentation techniques.

Diffusion models, unlike GANs, do not rely on adversarial training. Instead, they model the data distribution by reversing a diffusion process, which starts from the data and adds noise at each step until it reaches a known prior distribution. Despite the fact that diffusion models have demonstrated superior performance over GANs in terms of image quality and other metrics, no significant research has been conducted to investigate their performance in complex applications such as plant disease synthesis. In this study, we delve into the principles of diffusion technology, contrasting its methodology and performance with state-of-the-art GAN solutions. We examine the guided inference models of GANs, named InstaGAN, and compare it with RePaint, a diffusion-based model. Our findings reveal that the diffusion model demonstrates superior quality in data augmentation against GAN-based solutions. This study offers valuable insights into the potential of diffusion models for data augmentation in plant disease detection, paving the way for future research in this promising field.

## 4 Methodology

The methodology section provides a comprehensive overview of the techniques and algorithms employed in this research. It includes the principles, architecture, and mathematical foundations of the methods under investigation, namely InstaGAN and RePaint. The section also delves into the principles of Denoising Diffusion Probabilistic Models (DDPM), which form the basis of the RePaint method. Understanding these methodologies is essential for replicating the research and building upon the findings.

### 4.1 InstaGAN

InstaGAN, or Instance-aware Generative Adversarial Network, is a novel approach for unsupervised image-to-image translation. It is particularly effective in challenging cases where an image has multiple target instances, and the translation task involves significant changes in shape. The methodology of InstaGAN is divided into several key components and introduces several unique features.

#### 4.1.1 Instance level control

At its core, InstaGAN is a specialized GAN that emphasizes instance-aware image-to-image translation. It uses binary instance masks to guide the transformation process, allowing for targeted modifications within specific instances. This control is achieved through a specialized loss function that considers both the traditional GAN loss and an instance-level loss. In the context of plant science, masks can be used to target specific leaves or flowers for transformation, while leaving the rest of the image unchanged. To understand how InstaGAN accomplishes this, let’s explore its architecture, instance-level control, training losses, and the unique sequential mini-batch translation technique.

#### 4.1.2 InstaGAN architecture

It builds upon the CycleGAN framework by Zhu et al. (2017), inspired by conditional GANs by Mirza and Osindero (2014). It consists of two coupled generators *G_XY_*: *X*×*A* → *Y* ×*B* and *G_Y X_*: *Y* ×*B* → *X*×*A*, and adversarial discriminators *D_X_*: *X* × *A* → {‘X’, ‘not X’} and *D_Y_*: *Y* × *B* → {‘Y’, ‘not Y’}. These generators play a pivotal role in the translation process. Notably, *G_XY _* transforms healthy leaf images *X* into their corresponding diseased versions *Y*, while *G_Y X _* performs the inverse operation, converting disease leaf images *Y* back to healthy ones *X*. Furthermore, these generators are responsible for the reconstruction of masks on both sides, where *A* represents binary masks for healthy leaves, and *B* represents binary masks for disease-infected leaves. This level of control is crucial for simulating plant diseases accurately. For example, you can target specific leaves or parts of leaves for transformation, leaving the rest of the image unchanged, which is essential for creating realistic synthetic data. By having generators responsible for mask reconstruction, InstaGAN ensures that the transformed images align with the corresponding masks, enhancing the accuracy of synthetic data generation. The cycle consistency ensures that translated images can be transformed back to their original state. This property helps maintain image quality and realism during the translation process.

The leaf image representation *h_GX_* as formulated in Equation 1 and the n-th instance mask representation $h_{GA}^{n}$ in Equation 2 in the generator *G* are presented below:


(1) hGX(x,a)=[fGX(x);∑i=1N fGA(ai)]

(2) hGAn(x,a)=[fGX(x);∑i=1N fGA(ai);fGA(an)]

*f_GX _* function extracts features from the healthy leaf image *x* and *f_GA_* extracts features from the binary mask *a_i_*.

The discriminator *D*’s representation, which is permutation-invariant to the instances, is formulated in Equation 3.


(3) hDX(x,a)=[fDX(x);∑i=1N fDA(ai)].

**Figure 1:**
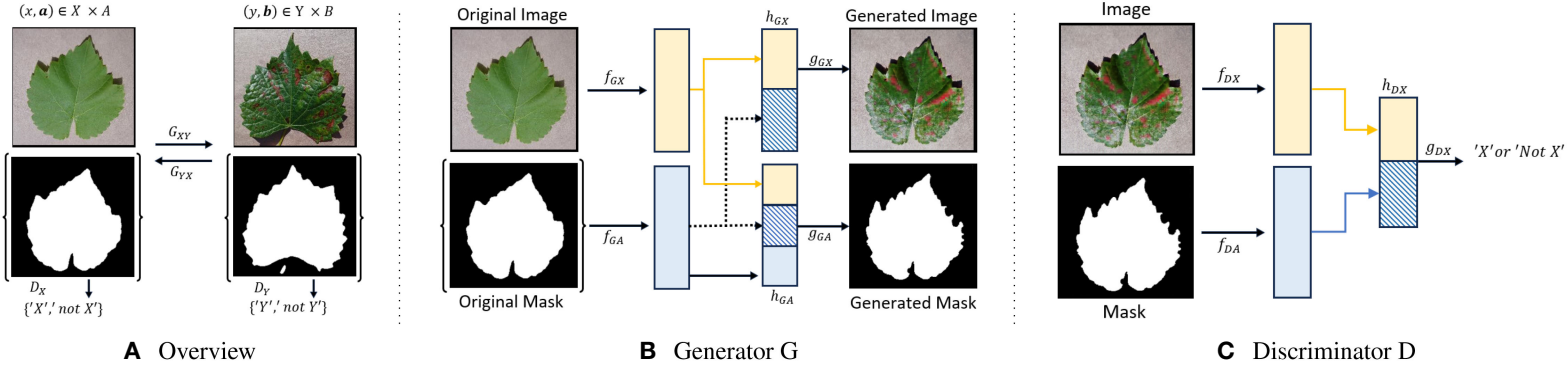
Overview, Generator, and Discriminator of InstaGAN Architecture. **(A)** Provides an overview of the image-to-image translation process, **(B)** illustrates the generator responsible for transforming healthy leaf images into disease-infected counterparts, and **(C)** showcases the discriminator’s role in distinguishing between generated and real images.

In **Figure 1**, an overview of the InstaGAN architecture is presented, illustrating its key components. The figure also provides a visual representation of both the generator and discriminator networks, offering insights into the underlying structure of InstaGAN.

#### 4.1.3 Training losses

The training process of InstaGAN incorporates several critical loss components, each serving a specific purpose in guiding the model’s learning. The GAN loss leverages the adversarial nature of GANs to encourage the generated images to be indistinguishable from real images in the target domain. Specifically, InstaGAN utilizes Least Square GAN by Mao et al. (2017) to ensure stable training, sharper image quality, and reduced mode collapse by improving gradient behavior and discriminator performance. It consists of two terms as shown in Equation 4. One term penalizes the difference between the discriminator’s prediction for real images. The other term penalizes the discriminator’s predictions for the translated images.


(4) $$L_{LSGAN}=\;{\left(D_{X}\left(x,a\right)\;-\;1\right)}^{2}+D_{X}{\left(G_{YX}\left(y,b\right)\right)}^{2}+\;{\left(D_{Y}\left(y,b\right)\;-\;1\right)}^{2}+D_{Y}{\left(G_{XY}\left(x,a\right)\right)}^{2}$$

The Cycle-Consistency Loss *L_cyc_* as introduced by CycleGAN is presented in Equation 5. *L_cyc_* is essential for maintaining the integrity of images throughout the translation process. This loss term measures the difference between the reconstructed images *G_YX_* (*G_XY_* (*x, a*)) and *G_XY_* (*G_Y X_* (*y, b*)) and their corresponding input images (*x,a*) and (*y,b*).


(5) $$L_{cyc}=\;∥G_{Y\;X}\left(G_{XY}\left(x,a\right)\right)\;-\;\left(x,a\right){∥}_{1}+\;∥G_{XY}\left(G_{YX}\left(y,b\right)\right)\;-\;\left(y,b\right)∥$$

Identity Mapping Loss *L_idt_* was also introduced by CycLeGAN. *L_idt_* as presented in Equation 6 measures the difference between the translated images *G_XY_* (*y,b*) and *G_YX_* (*x,a*) and their corresponding original images (*y,b*) and (*x,a*). This ensures that images do not lose their essential characteristics during the translation process.


(6) $$L_{idt}=\;∥G_{XY}\left(y,b\right)\;-\;\left(y,b\right){∥}_{1}+\;∥G_{YX}\left(x,a\right)\;-\;\left(x,a\right)∥$$

Context Preserving Loss *L_ctx_* as proposed originally for InstaGAN encourages the network to focus on translating instances while preserving the background context. This loss term is computed based on weighted differences between the translated and original images, as shown in Equation 7 taking into account the binary masks (*a,b* ^′^) and (*b,a* ^′^) that define which regions are translated.


(7) $$L_{ctx}=\;∥w\left(a,b^{\prime }\right)\;⊙\;\left(x-y^{\prime }\right){∥}_{1}+\;∥w\left(b,a^{\prime }\right)\;⊙\;\left(y-x^{\prime }\right)∥$$

The combination of these loss components denoted as *L_InstaGAN_* is presented in Equation 8. Hyperparameters *λ_cyc_*, *λ_idt_*, *λ_ctx_* are used to control the influence of each loss term, allowing for fine-tuning and balancing during the training process.


(8) $$L_{InstaGAN}=L_{LSGAN}+\lambda_{cyc}L_{cyc}+\lambda_{idt}L_{idt}+\lambda_{ctx}L_{ctx}$$

InstaGAN introduces a sequential mini-batch translation technique to handle an arbitrary number of instances without increasing GPU memory. The sequential version of the training loss is presented in Equation 9.


(9) $$L_{InstaGAN-SM}=\sum_{m=1}^{M}L_{LSGAN}\left(\left(x,a\right),\left({y^{\prime }}_{m},{b^{\prime }}_{1}:m\right)\right)+L_{content}\left(\left(x_{m},a_{m}\right),\left({y^{\prime }}_{m},{b^{\prime }}_{m}\right)\right)$$

where $L_{\mathrm{content}}=\lambda_{cyc}L_{cyc}+\lambda_{idt}L_{idt}+\lambda_{ctx}L_{ctx}$ .

However, for the current Plant Village dataset, which predominantly consists of images with a single leaf instance per image, the application of the sequential mini-batch technique may not be imperative. Nonetheless, it’s worth noting that this technique remains a valuable tool in our arsenal and could be considered for future datasets or scenarios where images contain multiple leaf instances per image, offering efficient training options in such cases.

### 4.2 RePaint

RePaint introduces a powerful inpainting approach, free-form inpainting, which involves adding new content to an image based on arbitrary binary masks. Unlike existing methods that struggle with generalization to unseen mask types and tend to produce simple textural extensions, RePaint presents a novel solution that leverages Denoising Diffusion Probabilistic Models (DDPM) to handle extreme masks effectively.

The core idea behind RePaint is to utilize a pretrained unconditional DDPM as the generative prior, enhancing its versatility and capability to generate high-quality inpainted images. To achieve this, the reverse diffusion iterations are modified to condition the generation process using the information provided by the input image. Importantly, RePaint achieves these improvements without altering or conditioning the original DDPM network, ensuring that it can produce diverse and top-quality output images regardless of the inpainting form. RePaint holds significant promise for enhancing our synthetic leaf data generation task, particularly in constructing disease-infected leaf samples from healthy leaf images as input when compared to InstaGAN. Unlike InstaGAN, which primarily focuses on image-to-image translation with an emphasis on instance-level control, RePaint’s strength lies in its ability to handle extreme and arbitrary binary masks. In our task context, RePaint can effectively simulate various disease patterns on healthy leaves, providing a more diverse and adaptable approach.

#### 4.2.1 Denoising diffusion probabilistic models

The DDPM learns a distribution of images given a training set. During training, DDPM methods define a diffusion process that transforms an image *x*_0_ to white Gaussian noise *x_T_* ∼ *N*(0,1) in *T* time steps. The forward direction is given by Equation 10.


(10) $$q(x_{t}|x_{t-1})\;=\;N(x_{t};\sqrt{1\;-\beta_{t}}x_{t-1},\beta_{t}I)$$

The sample *x_t_* is obtained by adding independent and identically distributed Gaussian noise with variance *β_t_* at timestep *t* and scaling the previous sample *x_t_*_−1_ with $\sqrt{1-\beta_{t}}$ according to a variance schedule.

The inference process works by sampling a random noise vector *x_T _* and gradually denoising it until it reaches a high-quality output image *x*_0_. This reverse process in Equation 11 is modeled by a neural network that predicts the parameters *µ_θ_* (*x_t_,t*) and Σ*_θ_* (*x_t_,t*) of a Gaussian distribution:


(11) $$p_{\theta }(x_{t}{-}_{1}|x_{t})\;=\;N(x_{t}{-}_{1};{µ}_{\theta }\left(x_{t},t\right),\Sigma_{\theta }\left(x_{t},\;t\right))$$

**Figure 2:**
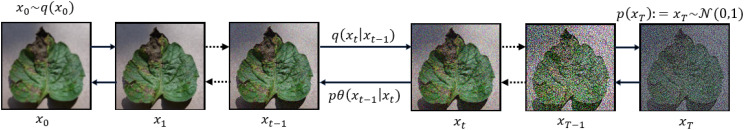
Overview of denoising diffusion probabilistic models.

Both forward and reverse diffusion processes presented by Equation 10 and 11 are illustrated in **Figure 2**. The learning objective is to predict the cumulative noise *ϵ*_0_ that is added to the current intermediate image *x_t_*. Therefore the objective is derived by considering the variational lower bound, leading to the following simplified training objective given in Equation 12:


(12) $$L_{\text{simple}}=E_{t,x_{0},\epsilon }\left[{\left\|\epsilon -\epsilon_{\theta }\left(x_{t},t\right)\right\|}^{2}\right]$$

By using the independence property of the noise added at each step, we can calculate the total noise variance as ${\bar{\alpha }}_{t}=\prod_{s=1}^{t}\left(1-\beta_{s}\right)$ . The reverse transition step in Equation 10 can be re-written as a single step as given below in Equation 13


(13) $$q(x_{t}|x_{0})=N\left(x_{t};\sqrt{{\bar{a}}_{t}}x_{0},\left(1-{\bar{a}}_{t}\right)I\right)$$

#### 4.2.2 Inpainting process

**Figure 3:**
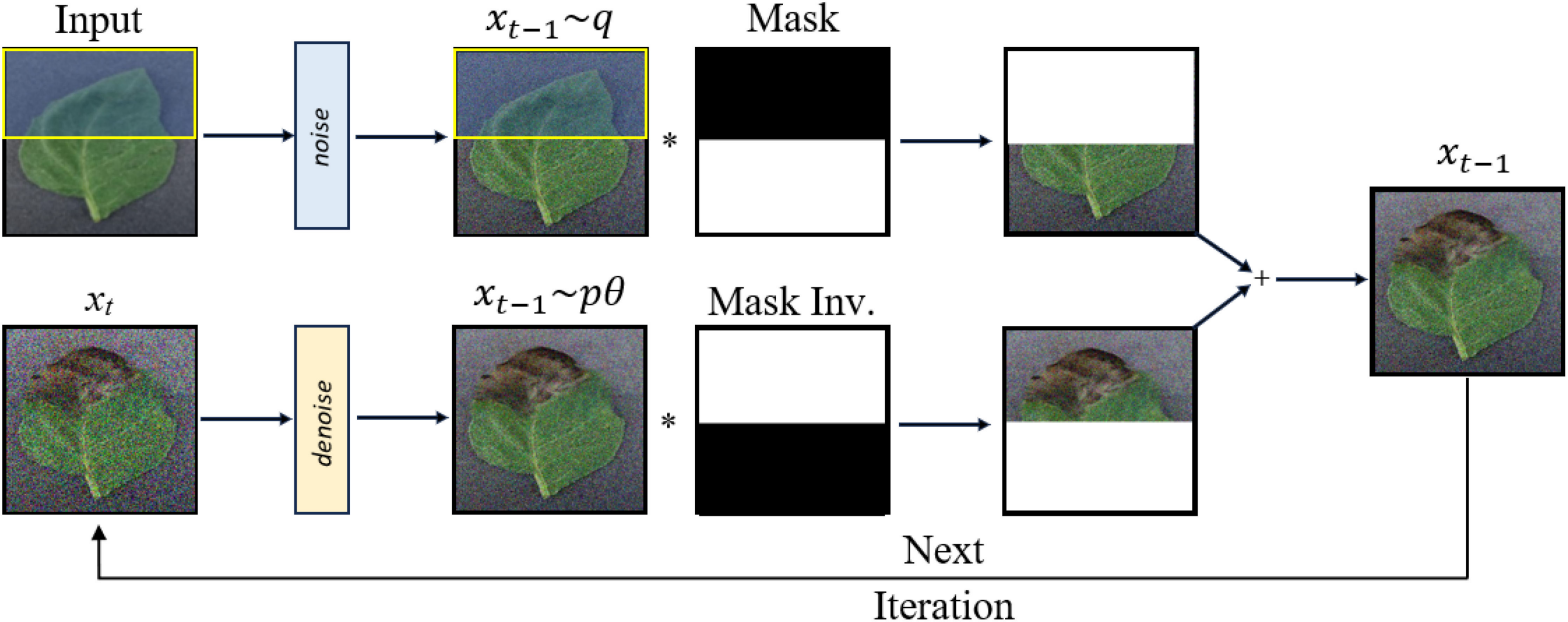
Overview of RePaint architecture.


In RePaint, masks are used to guide the inpainting process as shown in **Figure 3**. They define the regions where the image needs to be reconstructed. By altering the denoising steps of DDPM and sampling the unmasked regions, RePaint achieves high-quality inpainting. This can be likened to completing a puzzle where certain pieces are missing. The goal of inpainting is to predict missing pixels of an image using a mask region as a condition. The reverse step in the approach is given by Equation 16:

(14) $$x_{t-1}^{known}∼N\left({\sqrt{\bar{\alpha }}}_{t}x_{0},\left(1-{\bar{\alpha }}_{t}\right)I\right)$$

(15) $$x_{t-1}^{unknown}∼N\left({µ}_{\theta }\left(x_{t},t\right),\;\Sigma_{\theta }\left(x_{t},t\right)\right)$$

(16) $$x_{t-1}=m⊙x_{t-1}^{known}+\left(1-m\right)⊙x_{t-1}^{unknown}$$

Here ground truth image is denoted as *x*, the unknown pixels are denoted as *m* ⊙ *x*, and the known pixels as (1−*m*)⊙*x*. When this method is directly applied it is observed that the content type matches only with the known regions in the current image. The inpainted region may match the neighboring region and it may result in semantically incorrect regions. The resulting image may not be harmonizing well with the remaining image. DDPM is trained to generate an image that lies within a data distribution and tries to produce consistent structures. RePaint uses this quality of DDPM by diffusing the output *x_t_*_−1_ back to *x_t_*. The resulting $x_{t}^{unknown}$ better harmonizes with $x_{t}^{known}$ and contains conditional information from it.

### 4.3 Comparison between InstaGAN and RePaint

- **Principles**: InstaGAN uses adversarial training, while RePaint uses denoising diffusion.
- **Utilization of Masks**: InstaGAN uses masks for instance-level control, while RePaint uses them for guided inpainting.
- **Applications**: InstaGAN is suitable for instance-level transformations, while RePaint is designed for image inpainting.
- **Complexity**: InstaGAN involves a more complex adversarial training process, while RePaint focuses on a simpler, gradual denoising process.

## 5 Dataset

### 5.1 PlantVillage dataset

The PlantVillage dataset is a comprehensive collection of leaf images that are labeled with 38 different disease categories or as healthy. It was created to facilitate research in automated plant disease diagnosis and classification. The dataset consists of over 54,000 images, covering 14 crop species and 26 diseases, making it one of the largest publicly available datasets of its kind Hughes and Salathé (2015).

The images in the PlantVillage dataset are collected under controlled conditions, ensuring consistent lighting and background. This allows for a more accurate evaluation of computer vision models designed to recognize plant diseases. The dataset includes a wide variety of leaf diseases, ranging from fungal and bacterial infections to viral and nutrient deficiencies. The diversity of diseases and the inclusion of healthy leaves provide a robust and representative sample for training and evaluating machine learning models.

**Figure 4:**
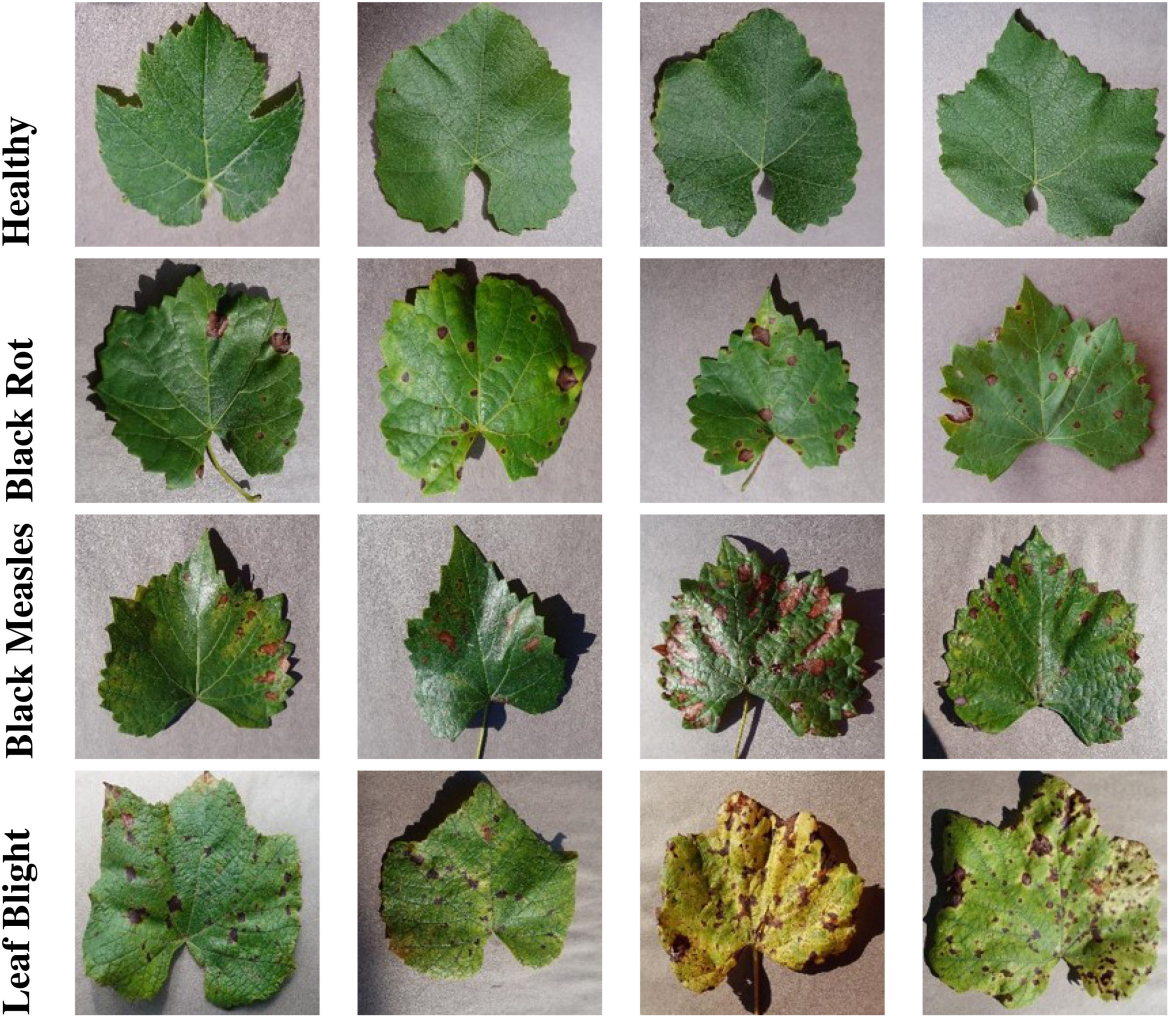
Sample images of grape leaves from the Plant Village dataset, showcasing different disease classes.

**Figure 5:**
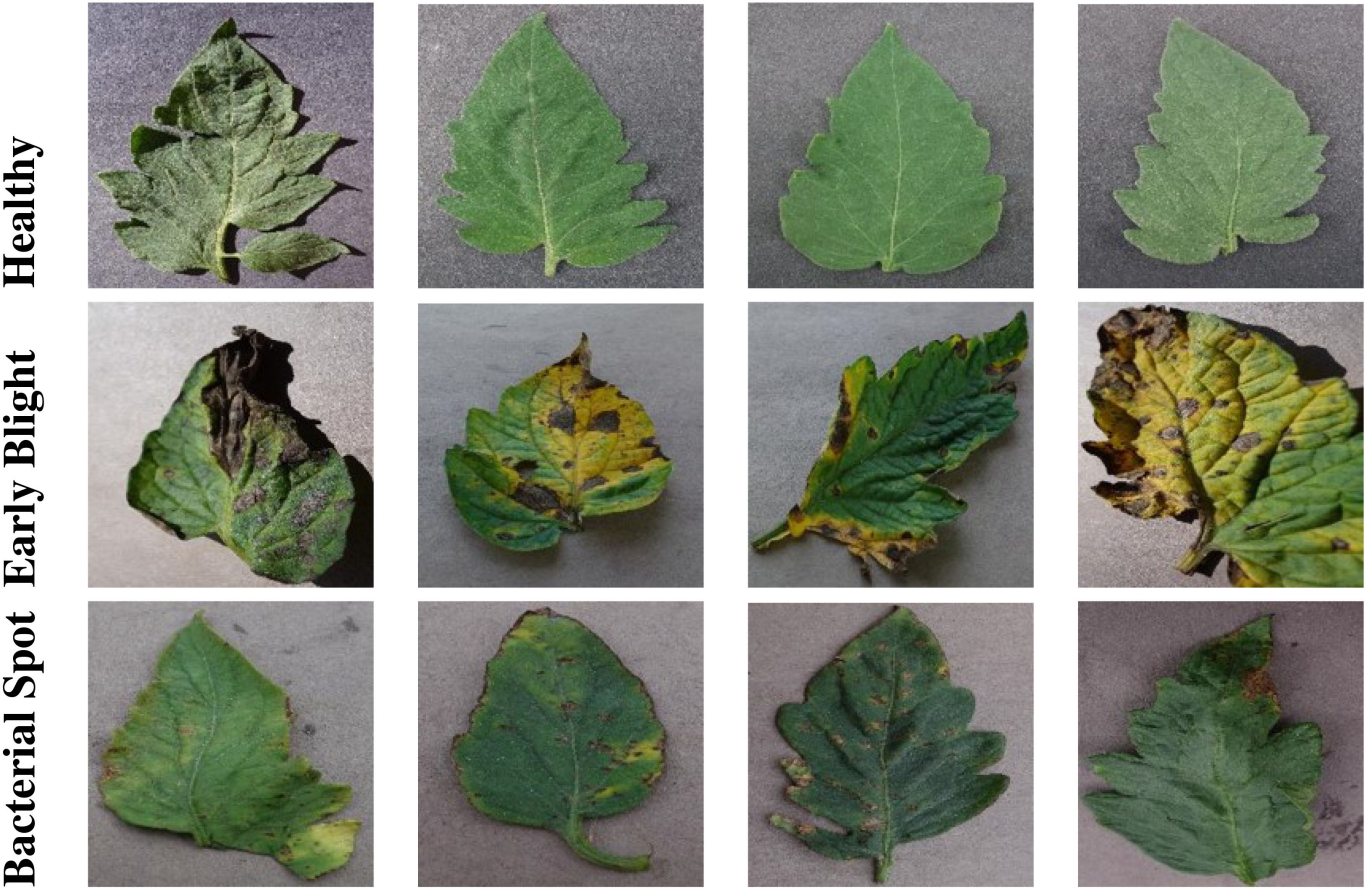
Sample images of tomato leaves from the Plant Village dataset, showcasing different disease classes.

**Figures 4**, **5** show sample images from the selected classes for grape and tomato leaves, respectively. For grape leaves, the images represent healthy leaves, black rot, black measles, and leaf blight, as shown in **Figure 4**. For tomato leaves, the images represent healthy leaves, early blight, and bacterial spot, as depicted in **Figure 5**. These images highlight the diversity and complexity of the leaf diseases within the dataset, emphasizing the variations in symptoms and visual characteristics for different disease classes.

The PlantVillage dataset has been instrumental in advancing the field of plant disease detection and classification. It has been used in numerous research studies to develop and evaluate machine learning models for automated plant disease diagnosis. By providing a standardized and publicly available resource, the PlantVillage dataset continues to drive innovation and progress in the field of agricultural technology.

A significant number of research publications have reported results specifically on tomato and grape leaf images. These two crops have been the subject of extensive study in the field of plant disease detection and classification. By focusing on these two crops, we align our work with existing research, allowing for a fair and meaningful comparison with other published methods. There for our experiments, we selected 9 disease classes of tomato leaves and 3 disease classes of grape leaves, along with healthy classes for both types of plants. **Table 1** provides detailed statistics for the selected classes from the PlantVillage dataset. It includes the number of images for each disease class and the healthy class for both tomato and grape leaves.

**Table 1:** Statistics for the selected classes from the PlantVillage dataset.

Dataset No	Plant Type	Diseases Name	Number of Images
1	Tomato	Late Blight	1000
2	Tomato	Early Blight	800
3	Tomato	Septoria Leaf Spot	700
4	Tomato	Target Spot	600
5	Tomato	Mosaic Virus	500
6	Tomato	Yellow Leaf Curl Virus	400
7	Tomato	Spider Mites	300
8	Tomato	Leaf Mold	200
9	Tomato	Bacterial Spot	100
10	Grape	Black Rot	500
11	Grape	Esca (Black Measles)	400
12	Grape	Leaf Blight	300
13	Tomato	Healthy	1200
14	Grape	Healthy	600
1 ∼ 9,13	Tomato	Total	5800
10 ∼ 12,14	Grape	Total	1800
1 ∼ 14	Total	Total	7600

The selected classes from the PlantVillage dataset provide a robust and representative sample for evaluating the performance of InstaGAN and RePaint. By focusing on specific disease classes and including healthy leaves, we ensure a fair and comprehensive comparison that reflects the real-world challenges of plant disease detection and transformation.

### 5.2 Mask data preparation

The efficacy of our image generation models, InstaGAN and Repaint, critically hinges upon the accessibility and quality of segmentation masks. These masks assume a pivotal role as guiding constructs in the generative processes, facilitating the models in the precise localization and transformation of specific regions within leaf imagery. In this subsection, we detail the meticulous procedure underpinning the preparation of segmentation masks for our dataset.

#### 5.2.1 Leaf segmentation masks for InstaGAN

The segmentation process in the InstaGAN framework is essential for precisely identifying and isolating regions of interest within leaf images. These regions are then used to guide the generative process for the transformation of healthy leaf images into their corresponding diseased versions. To create accurate segmentation masks, a dataset comprising a diverse set of leaf images was employed. As mentioned in the previous section, the dataset consisted of a total of 7100 leaf images belonging to 14 classes. Of these, 1033 pairs of images and their corresponding manually annotated masks were utilized for training the segmentation model, while an additional 230 pairs were reserved for evaluation purposes. It’s worth noting that the training dataset was deliberately designed to include images from various classes, ensuring the model’s ability to generalize across different leaf types and disease symptoms.

The segmentation network architecture is based on the U-Net framework, which is renowned for its effectiveness in image segmentation tasks. The U-Net architecture is particularly suited for its ability to capture fine-grained information while preserving spatial details. The backbone of the segmentation model utilized in this research is based on ResNet-50, a well-established deep learning architecture known for its feature extraction capabilities. This choice of backbone enhances the model’s ability to capture intricate details within the leaf images. During the training phase, the segmentation model learned to generate precise masks that delineate the boundaries of leaves in the images. The manually annotated masks from the training dataset served as ground truth labels for supervising the model’s learning process. This supervised training process enabled the segmentation model to understand the intricate patterns and shapes of leaves across various classes.

**Figure 6:**
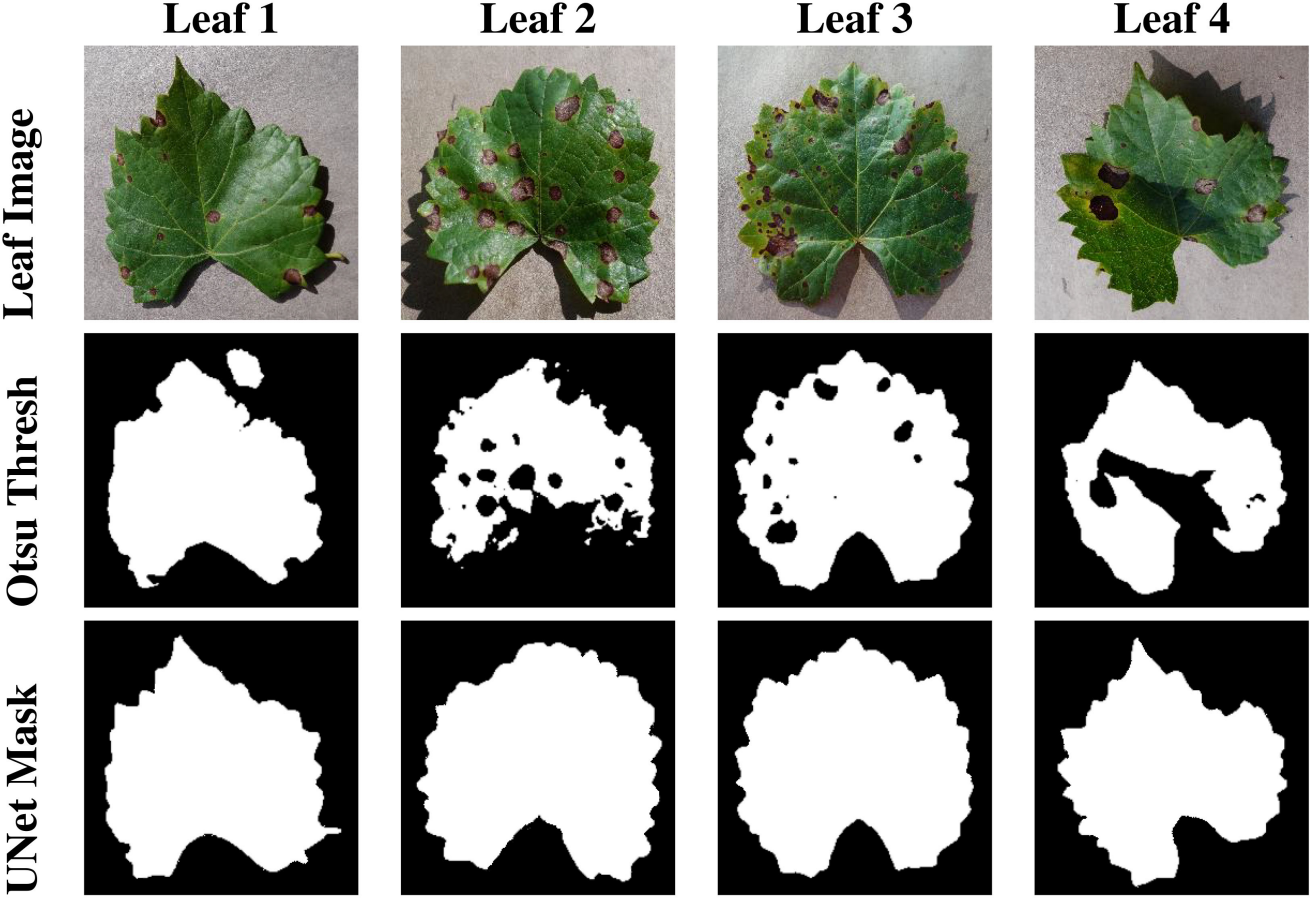
This figure presents the segmentation masks generated by Otsu Thresholding, and U-Net model.

Recognizing the challenges associated with manual annotation, an alternative method was explored. Otsu thresholding, a simple yet effective technique, was applied to some images to extract leaf masks automatically. While Otsu thresholding performed well on certain images, it encountered limitations when applied to a larger and more diverse dataset. The shortcomings included the inclusion of unwanted background regions and shadows in the final mask. Moreover, it failed to accommodate various disease symptoms, resulting in the omission of relevant details from the leaf mask. The qualitative results are presented in **Figure 6**.

To quantitatively assess the performance of the segmentation process, the mean Intersection over Union (mIOU) metric was computed. This metric provides a quantitative measure of the overlap between the predicted masks and the ground truth masks. The results of this evaluation are presented in 2, offering insights into the accuracy and effectiveness of the segmentation model. Our evaluation results clearly demonstrate the effectiveness of the segmentation models. The UNet model achieved an impressive mIOU score of 97.43%, indicating its ability to accurately delineate leaf regions within images. This high level of accuracy is crucial for guiding the generative process of InstaGAN effectively. While the Otsu thresholding also performed well, it exhibited slightly lower mIOU scores in comparison as presented in **Table 2**. This method, although proficient, did not match the precision achieved by our custom-trained U-Net on our dataset.

**Table 2:** Segmentation model performance.

Model	mIOU
Otsu Threshold	75.36%
U-Net (Backbone ResNet 50)	97.43%

#### 5.2.2 Masks for RePaint

Repaint utilizes masks to guide the diffusion process, similar to our GAN-based InstaGAN. However, there are several key distinctions in how Repaint operates. One crucial difference is that Repaint requires inverted masks. In this context, the white regions of the mask are used to evaluate contextual information, while the black regions are regenerated. This inverted mask approach is fundamental to the unique functioning of Repaint. When applying inverted versions of the masks created in the previous section, we encountered a challenge known as the “ghost leaf problem.” This problem manifests as the outline of the input leaf image being filled with background texture, and within this outline, a smaller leaf appears. The output image seems to contain two leaves: one leaf with the desired disease symptoms but entirely different from the input image, and another larger leaf that matches the outline of the input leaf but has become transparent.

The root cause of this problem lies in the difference in image generation approaches used by InstaGAN and Repaint. InstaGAN regenerates the masked region with desired features, such as disease symptoms. In contrast, Repaint gradually adds noise to the region until it’s entirely filled with noise and then regenerates the region based on local context, i.e., other parts of the leaf. However, because we masked the entire leaf region, there was insufficient contextual information to guide the generation process, except for a very thin outline of the leaf unaccounted for by the segmentation mask. As a result, Repaint attempted to fill the masked region with an entire image, including the background and a random leaf with desired features.

To address the ghost leaf problem, we modified the segmentation masks by dilating them, leaving more area along the boundaries of the leaf. This adjustment aimed to provide enough information for Repaint to regenerate the input leaf image with the desired disease features. This solution proved effective, as Repaint was then able to utilize the bordering area of the leaf to generate the remaining portions with the desired diseased features. However, this method introduced a drawback. Since the bordering area of the input image with the healthy leaf was not masked, it was never regenerated with disease symptoms. This could result in a significant data bias in the synthetic dataset, as many diseases affect the leaf edges more than the central regions.

Through experimentation, it was deduced that Repaint does not necessarily require a well-bordered region of the leaf to generate new leaf images. Any part of the leaf image can assist the diffusion process in completing the remaining part with the desired disease symptoms. For instance, if half of the leaf is masked while the rest is unmasked, Repaint generates a seamlessly blended version of the remaining leaf with the desired disease symptoms. Importantly, since there is no constraint of a close boundary, the Repaint model is free to create versions of the leaf with different boundaries and shapes than the input image. This diversity enhances the novelty of the results, including the creation of leaf versions not present in the input data. Disease symptoms are generated in the newly generated regions of the leaf, encompassing the boundary areas and edges.

**Figure 7:**
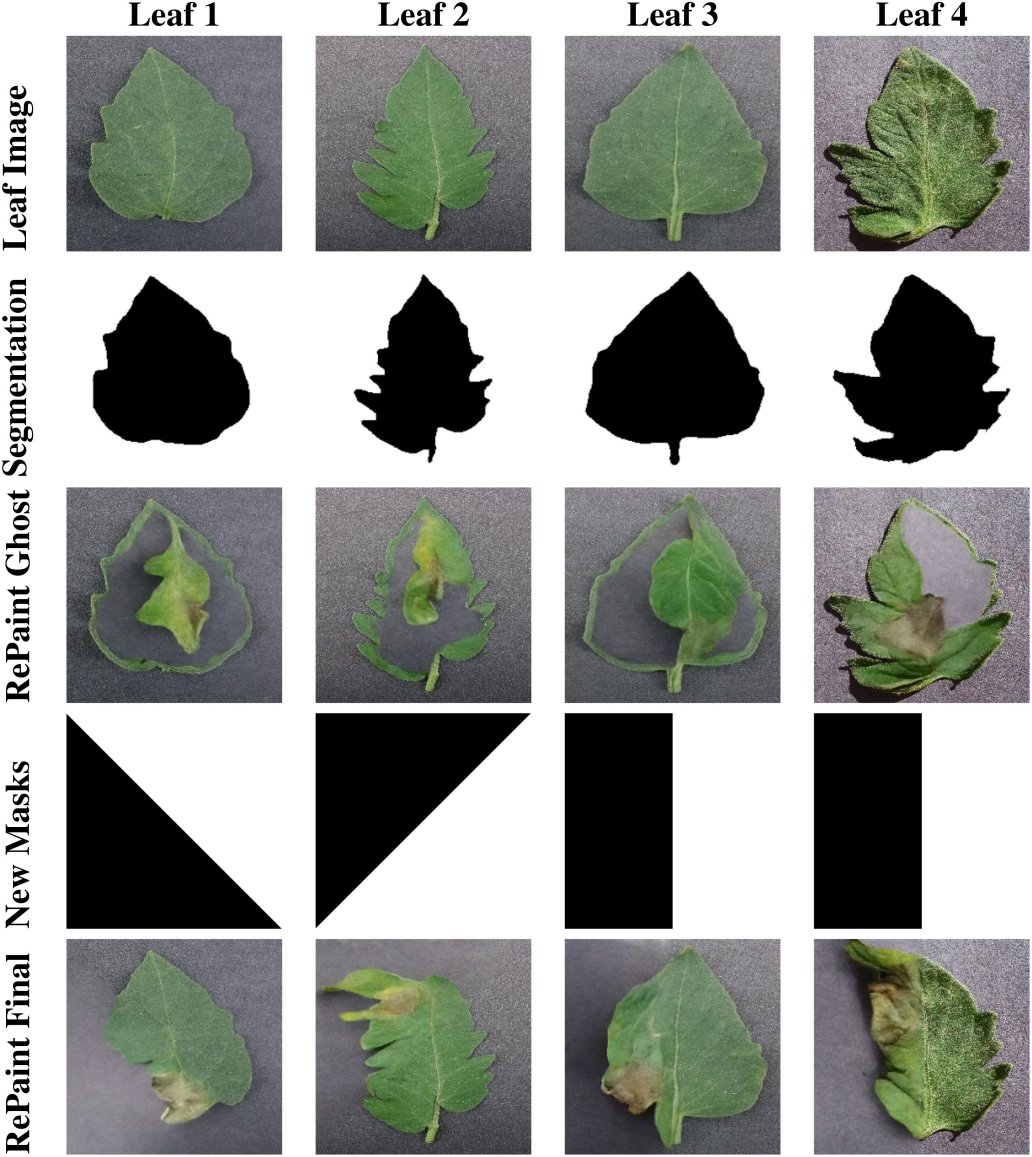
This figure displays the stages of RePaint’s image generation process. Row 1 shows healthy leaf images, Row 2 reveals the corresponding segmentation masks, and Row 3 displays the output of Repaint when using segmentation masks for guidance. Row 4 presents the split masks, while Row 5 showcases the results achieved by Repaint when utilizing these split masks for image generation. This comparison highlights the effectiveness of different mask strategies in RePaint’s generative capabilities.

To ensure a balanced representation of synthetic disease symptoms across all parts of the leaf, various versions of simple masks were created and randomly applied at a uniform distribution. The results of this approach were remarkably positive, providing a diverse set of synthetic disease symptoms. Sample masks and output images are illustrated in **Figure 7**, showcasing the effectiveness of our mask generation strategy in Repaint.

## 6 Performance measures

In the field of image generation, synthesis, and augmentation, a variety of evaluation metrics are commonly employed to assess the quality and effectiveness of the methods. In this research, we considered a comprehensive set of evaluation metrics to assess the effectiveness of the proposed methods. These metrics provide a quantitative analysis of the performance, capturing various aspects of image quality, similarity, and statistical properties. However, the evaluation of generative AI models has resulted, in the introduction of new metrics that address the limitations of previous methods and cater to advanced applications. Therefore, we utilize a subset of these metrics that are particularly relevant to our study, while acknowledging that some commonly used metrics may not be as applicable in our context.

Below, we introduce each metric, explaining its working principles, and reflecting on their development and significance in the field.

### 6.1 Peak signal-to-noise ratio

PSNR was one of the early metrics used to measure the quality of a reconstructed image compared to the original. However, it primarily focuses on pixel-level differences and may not capture perceptual quality. The equation for PSNR is:


(17) $$PSNR=20\cdot \log_{10}\left(\frac{MAX_{I}}{\sqrt{MSE}}\right)$$

### 6.2 Structural similarity index

To address the limitations of PSNR, SSIM was introduced to assess the perceptual similarity between two images. However, in some contexts, SSIM may not be as pertinent. The equation for SSIM is:


(18) $$SSIM\left(x,y\right)=\frac{\left(2\mu_{x}\mu_{y}+c_{1}\right)\left(2\sigma_{xy}+c_{2}\right)}{\left(\mu_{x}^{2}+\mu_{y}^{2}+c_{1}\right)\left(\sigma_{x}^{2}+\sigma_{y}^{2}+c_{2}\right)}$$

### 6.3 Inception score

IS was developed to measure both the quality and diversity of generated images, addressing the need for a more comprehensive evaluation. Higher IS values indicate better performance. The equation for IS is:


(19) $$IS=\;exp({\text{E}}_{x}D_{KL}\left(p\left(y|x\right)\|p\left(y\right)\right))$$

### 6.4 Fréchet inception distance

FID was introduced to overcome the limitations of IS by measuring the statistical similarity between real and generated images. Lower FID values indicate better quality and similarity. The equation for FID is:


(20) $$FID=\;\|{µ}_{real}-{µ}_{fake}|{|}^{2}+Tr(\Sigma_{real}+\;\Sigma_{fake}-\;2{\left(\Sigma_{real}\Sigma_{fake}\right)}^{0.5})$$

### 6.5 Kernel inception distance

KID further advanced the field by comparing the distribution of Inception features between real and generated images. Unlike FID, KID is unbiased and does not require a large number of samples. The equation for KID is:


(21) $$KID=\text{E}\left[\kappa \left(\left(x_{i},y_{j}\right)\right)\right]$$

### 6.6 Effectiveness in this research

In our study, we focused on FID and KID for detailed comparison between InstaGAN and RePaint, reflecting the complexity and nuances of our research in image generation, synthesis, and augmentation. FID was also used as the primary metric for evaluating our methods against existing works, given its widespread adoption in the literature. By carefully selecting and employing these metrics, we ensure a rigorous and targeted assessment of performance, capturing the evolution and advancements in the field.

## 7 Experimental settings

### 7.1 InstaGAN settings

InstaGAN was configured with the following key parameters for the experiments:

- **Batch Size**: Set to 1, controlling the number of training samples processed simultaneously. A smaller batch size was chosen to allow for more frequent updates and to fit the model into GPU memory.
- **Image Sizes**: Load size of 220x220 and fine size of 200x200 were used for scaling and cropping. These sizes were selected to preserve the details of the images while reducing computational complexity.
- **Number of Filters**: 64 filters in the first convolution layer for both generator and discriminator, providing a balance between model complexity and computational efficiency.
- **Learning Rate**: Set to 0.0002, with a decay after 100 iterations, allowing the model to converge smoothly without overshooting the optimal solution.
- **Dropout**: Disabled, to prevent overfitting and ensure stable training.
- **Normalization**: Instance normalization was used to normalize the activations within a feature map, improving the training stability.
- **Data Augmentation**: Random flipping and resizing with cropping were applied to increase the diversity of the training data and enhance the model’s generalization ability.

### 7.2 RePaint settings

RePaint was configured with the following key parameters for the experiments:

- **Attention Resolutions**: Set to 32, 16, 8, defining the resolutions for attention mechanisms. These resolutions allow the model to capture different levels of details in the images.
- **Diffusion Steps**: 4000 steps were used, controlling the number of diffusion steps in the process. A higher number of steps enables more refined image generation.
- **Number of Channels**: 128 channels were used, defining the complexity of the model and allowing it to capture intricate patterns.
- **Learning Rate Kernel Standard Deviation**: Set to 2, controlling the adaptiveness of the learning rate during training.
- **Use of 16-bit Precision**: Enabled, to reduce memory consumption and accelerate training without significant loss of accuracy.
- **Image Size**: 256, defining the size of the input images, chosen to retain sufficient details while managing computational resources.

These settings were carefully chosen to align with the specific requirements of the experiments and to ensure optimal performance of both InstaGAN and RePaint models. The selection of parameters reflects a balance between model complexity, computational efficiency, and the ability to capture the underlying patterns in the data.

## 8 Results

### 8.1 Qualitative results

The qualitative analysis of the generated images for 12 distinct disease classes provides a comprehensive understanding of the performance of both InstaGAN and RePaint.

#### 8.1.1 Early blight in tomato leaves

Early blight in tomato leaves is characterized by concentric rings and dark spots, often leading to wilting and death of the plant. These intricate patterns may pose challenges for generative models, as capturing the precise shape and texture of the rings requires a high level of detail. **Supplementary Figure 1** illustrates the healthy tomato leaf images, their corresponding segmentation masks, and the generated images depicting early blight disease symptoms by both InstaGAN and RePaint. While InstaGAN’s outputs are commendable, RePaint significantly outperforms InstaGAN in capturing these complex symptoms.

#### 8.1.2 Late blight in tomato leaves

Late blight symptoms include intricate patterns and authentic appearance. **Supplementary Figure 2** presents how RePaint excels in capturing these patterns, overcoming the challenge of detailed complexity. In contrast, while InstaGAN manages to simulate the disease’s presence, it may struggle to depict the fine details that make late blight unique.

#### 8.1.3 Tomato black spot

Tomato black spot disease manifests as dark, sunken lesions. The irregular shapes and varying sizes of the spots can be challenging for AI models to replicate accurately. **Supplementary Figure 3** presents the results for this disease, with RePaint’s generated images exhibiting a higher level of detail and quality compared to InstaGAN.

#### 8.1.4 Target spot in tomato leaves

Target spot symptoms are characterized by concentric rings and discolorations, making them a challenging task for generative models. **Supplementary Figure 4** showcases how RePaint excels in capturing the intricate details of target spot, reproducing the characteristic concentric rings and discolorations with high fidelity. While InstaGAN may simulate some ring-like patterns, it may not capture the full complexity of the disease’s appearance.

#### 8.1.5 Septoria leaf spot in tomato leaves

Septoria leaf spot symptoms involve precise spot patterns and discolorations. **Supplementary Figure 5** delves into how RePaint excels in replicating these patterns, providing an authentic portrayal of this complex disease. While InstaGAN may exhibit some spot-like effects, it may struggle to capture the full intricacy of the symptoms.

#### 8.1.6 Two spotted spider mites in tomato leaves

Two spotted Spider mites symptoms include distinctive patterns and discolorations. **Supplementary Figure 6** explores how RePaint excels in replicating these patterns, offering a convincing representation of the disease’s complexity. While InstaGAN may capture some aspects of the disease, it may not fully convey the intricate details that define spider mites two-spotted.

#### 8.1.7 Yellow leaf curl virus in tomato leaves

Yellow leaf curl virus symptoms involve leaf curling and yellowing, presenting a complex set of characteristics. **Supplementary Figure 7** explores how RePaint accurately reproduces the curling and yellowing of leaves, providing an authentic representation of the disease’s intricacies. While InstaGAN may simulate some aspects of the disease, it may struggle to convey the full complexity and nuances of yellow leaf curl virus symptoms.

#### 8.1.8 Mosaic virus in tomato leaves

Mosaic virus symptoms involve intricate mosaic patterns and discolorations. **Supplementary Figure 8** explores how RePaint accurately reproduces these patterns, delivering a convincing representation of the disease’s intricacies. While InstaGAN may simulate some aspects of the disease, it may not fully convey the level of detail and realism achieved by RePaint.

#### 8.1.9 Leaf mold in tomato leaves

Leaf mold symptoms involve challenging mold patterns. **Supplementary Figure 9** presents how RePaint adeptly reproduces these patterns, offering a convincing representation of the disease’s intricacy. InstaGAN, though attempting to emulate the disease style, may find it challenging to convey the nuanced details that define leaf mold.

#### 8.1.10 Grape Leaf black measle

Grape leaf black measle is characterized by dark spots with a complex pattern. Modeling such symptoms requires capturing both the geometry and texture of the affected areas, which can be challenging for deep learning models. **Supplementary Figure 10** showcases the ability of RePaint to synthesize these complex patterns, outperforming InstaGAN.

#### 8.1.11 Grape leaf black rot

Grape leaf black rot presents as dark, rotting areas with defined edges. The sharp transitions and consistent coloring of the rotting areas may pose difficulties for generative models. **Supplementary Figure 11** reveals similar trends between InstaGAN and RePaint, with RePaint’s images exhibiting a more refined portrayal.

#### 8.1.12 Grape leaf blight

Grape leaf blight involves subtle variations in color and texture, which can be particularly challenging for AI to reproduce accurately. **Supplementary Figure 12** presents the results for this disease, with RePaint demonstrating its superiority in generating images that closely resemble the actual appearance.

The qualitative analysis across all 12 disease classes underscores the remarkable performance of RePaint, especially in comparison to InstaGAN. While InstaGAN provides a reasonable approximation of the disease symptoms, RePaint’s ability to capture the intricate details sets it apart. These findings reinforce the potential of RePaint as a powerful tool in the field of precision agriculture.

### 8.2 Quantitative evaluation

The quantitative evaluation of the proposed methods, InstaGAN and RePaint, was conducted using the Fréchet Inception Distance (FID) and Kernel Inception Distance (KID) metrics. Both of these metrics are widely used for evaluating the quality of generated images, and it measures the statistical similarity between the real and generated distributions. Lower FID and KID values indicate better performance, as they signify that the generated images are more similar to the real ones.

The FID results for both methods on different disease classes of grape and tomato leaves are presented in **Table 3**. The results demonstrate that RePaint consistently outperforms InstaGAN across all the tested classes, achieving lower FID values.

**Table 3:** FID results for InstaGAN and RePaint on different disease classes.

Dataset No	Plant	Disease	InstaGAN	RePaint
1	Grape	Black rot	81.71	56.02
2	Grape	Esca (Black Measles)	105.89	68.83
3	Grape	Leaf blight (Isariopsis Leaf Spot)	155.25	82.30
4	Tomato	Bacterial spot	271.28	181.39
5	Tomato	Early blight	195.33	135.84
6	Tomato	Late Blight	212.47	143.62
7	Tomato	Septoria Leaf Spot	225.13	153.49
8	Tomato	Target Spot	203.78	138.94
9	Tomato	Mosaic Virus	287.56	196.72
10	Tomato	Yellow Leaf Curl Virus	228.94	156.08
11	Tomato	Spider Mites	259.63	178.21
12	Tomato	Leaf Mold	245.37	167.89
1 ∼ 3	Grape	Average	114.28	69.05
4 ∼ 12	Tomato	Average	236.61	161.35
1 ∼ 12	Total	Average	206.02	138.28

The results indicate that RePaint is more effective in capturing the underlying distribution of the real images, leading to more realistic and accurate synthetic images. The improvement in FID scores for RePaint over InstaGAN suggests that the diffusion-based approach of RePaint offers advantages in generating high-quality images for the specific task of plant disease image augmentation.

The Kernel Inception Distance (KID) results for both InstaGAN and RePaint methods across various plant diseases are presented in **Table 4**. These results demonstrate the effectiveness of RePaint, particularly for Grape diseases, where it consistently achieves lower KID scores. The performance on Tomato diseases also indicates the robustness and adaptability of RePaint across different plant types and diseases. The comparative analysis between InstaGAN and RePaint provides valuable insights into the strengths and weaknesses of both methods, contributing to the understanding of their applicability in plant disease image synthesis and augmentation.

**Table 4:** KID results for InstaGAN and RePaint on different disease classes.

Dataset No	Plant	Disease	InstaGAN	RePaint
1	Grape	Black rot	0.098 ( ± 0.002)	0.026 ( ± 0.001)
2	Grape	Esca (Black Measles)	0.081 ( ± 0.002)	0.035 ( ± 0.002)
3	Grape	Leaf blight (Isariopsis Leaf Spot)	0.161 ( ± 0.004)	0.046 ( ± 0.002)
4	Tomato	Bacterial spot	0.125 ( ± 0.002	0.104 ( ± 0.002)
5	Tomato	Early blight	0.064 ( ± 0.001)	0.057 ( ± 0.002)
6	Tomato	Late Blight	0.212 ( ± 0.005)	0.143 ( ± 0.003)
7	Tomato	Septoria Leaf Spot	0.225 ( ± 0.006)	0.153 ( ± 0.004)
8	Tomato	Target Spot	0.203 ( ± 0.005)	0.138 ( ± 0.003)
9	Tomato	Mosaic Virus	0.287 ( ± 0.007)	0.197 ( ± 0.004)
10	Tomato	Yellow Leaf Curl Virus	0.229 ( ± 0.006)	0.157 ( ± 0.004)
11	Tomato	Spider Mites	0.260 ( ± 0.007)	0.179 ( ± 0.004)
12	Tomato	Leaf Mold	0.245 ( ± 0.006)	0.168 ( ± 0.004)
1 ∼ 3	Grape	Average	0.1133 ( ± 0.0027)	0.0357 ( ± 0.00167)
4 ∼ 12	Tomato	Average	0.205 ( ± 0.0055)	0.144 ( ± 0.00267)
1 ∼ 12	Total	Average	0.1591 ( ± 0.0041)	0.08985 ( ± 0.00217)

## 9 Comparison with state-of-the-art methods

In the rapidly evolving field of generative models for plant disease image synthesis, it is essential to benchmark new methods against existing state-of-the-art techniques. This comparison provides insights into the relative strengths and weaknesses of different approaches, guiding future research and development. The following subsections present a detailed comparison of our proposed methods, InstaGAN and RePaint, with other leading methods, focusing on their performance in synthesizing images for Tomato and Grape Leaf diseases.

### 9.1 Comparison on tomato leaf diseases

**Table 5** presents a comparison of the Frechet Inception Distance (FID) scores for various methods applied to the PlantVillage dataset, focusing on Tomato crop diseases. The FID score is a widely used metric to measure the quality of generated images, with lower scores indicating higher similarity between the generated and real images.

**Table 5:** Comparison of FID scores with other publications.

Method	Dataset	Crop	Disease Classes	FID
WGAN	PlantVillage	Tomato	Healthy, Yellow leaf curl virus, Leaf mold, Spider mite	226.08
SAGAN	PlantVillage	Tomato	Healthy, Yellow leaf curl virus, Leaf mold, Spider mite	229.7233
MAGAN	PlantVillage	Tomato	Healthy, Yellow leaf curl virus, Leaf mold, Spider mite	220.69
HA+DMM	PlantVillage	Tomato	Healthy, Yellow leaf curl virus, Leaf mold, Spider mite	219.0633
MMDGAN	PlantVillage	Tomato	Healthy, Yellow leaf curl virus, Leaf mold, Spider mite	214.8867
InstaGAN	PlantVillage	Tomato	Late Blight, Early Blight, Septoria Leaf Spot, Target Spot, Mosaic Virus, Yellow Leaf Curl Virus, Spider Mites, Leaf Mold, Bacterial Spot	236.61
RePaint	PlantVillage	Tomato	Late Blight, Early Blight, Septoria Leaf Spot, Target Spot, Mosaic Virus, Yellow Leaf Curl Virus, Spider Mites, Leaf Mold, Bacterial Spot	**161.35**

Several methods, including WGAN, SAGAN, MAGAN, HA+DMM, and MMDGAN, were applied to the Tomato crop, focusing on 4 disease classes: Healthy, Yellow leaf curl virus, Leaf mold, and Spider mite. The FID scores for these methods range from 214.8867 (MMDGAN) to 229.7233 (SAGAN), indicating varying levels of performance in generating realistic images.

In contrast, InstaGAN and RePaint were applied to 9 different Tomato disease classes: Late Blight, Early Blight, Septoria Leaf Spot, Target Spot, Mosaic Virus, Yellow Leaf Curl Virus, Spider Mites, Leaf Mold, and Bacterial Spot. RePaint significantly outperforms InstaGAN with an FID score of 161.35 compared to InstaGAN’s score of 236.61. This highlights the superior performance of RePaint in generating high-quality images that closely resemble the real data.

This comparison provides valuable insights into the state-of-the-art methods in the field of generative models for plant disease image synthesis. It also emphasizes the effectiveness of RePaint, particularly in comparison to other leading methods, demonstrating its potential as a powerful tool for various applications in plant science and computer vision.

### 9.2 Comparison on grape leaf diseases

**Table 6** presents a comparison of the Frechet Inception Distance (FID) scores for various methods applied to the PlantVillage dataset, focusing on Grape Leaf diseases. The diseases considered in this comparison include Black Rot, Black Measles, and Leaf Blight.

**Table 6:** Comparison of FID scores on grape leaf diseases.

Method	Dataset	Subset	Classes	FID
DCGAN	PlantVillage	Grape Leaf	Black Rot, Black Measles, Leaf Blight	309.376
LeafGAN	PlantVillage	Grape Leaf	Black Rot, Black Measles, Leaf Blight	178.256
E-GAN	PlantVillage	Grape Leaf	Black Rot, Black Measles, Leaf Blight	112.563
InfoGAN	PlantVillage	Grape Leaf	Black Rot, Black Measles, Leaf Blight	178.13
WGAN	PlantVillage	Grape Leaf	Black Rot, Black Measles, Leaf Blight	121.31
LRGAN	PlantVillage	Grape Leaf	Black Rot, Black Measles, Leaf Blight	128.23
Fine Grained GAN	PlantVillage	Grape Leaf	Black Rot, Black Measles, Leaf Blight	72.73
InstaGAN	PlantVillage	Grape Leaf	Black Rot, Black Measles, Leaf Blight	114.28
RePaint (Diffusion)	PlantVillage	Grape Leaf	Black Rot, Black Measles, Leaf Blight	**69.05**

Several generative models, including DCGAN, LeafGAN, E-GAN, InfoGAN, WGAN, LRGAN, and Fine Grained GAN, were applied to the Grape Leaf subset. The FID scores for these methods range from 72.73 (Fine Grained GAN) to 309.376 (DCGAN), reflecting a wide range of performance levels.

InstaGAN and RePaint (Diffusion) were also applied to the same subset, with RePaint achieving the lowest FID score of 69.05, outperforming all other methods. This result emphasizes the effectiveness of RePaint, particularly in generating high-quality images of Grape Leaf diseases, and demonstrates its superiority over other leading methods.

## 10 Discussion

In this study, we introduced and evaluated two novel methods, InstaGAN and RePaint, for plant disease image synthesis. Our comprehensive comparison with state-of-the-art methods on both Tomato and Grape Leaf diseases revealed the superior performance of RePaint, particularly in generating high-quality images that closely resemble real data. The results of this study have several important implications. First, the effectiveness of RePaint demonstrates the potential of diffusion-based models in the field of generative models for plant science and computer vision. Second, the ability to synthesize realistic images of plant diseases can significantly enhance data augmentation techniques, providing a valuable tool for training more robust and accurate disease detection models. While the findings are promising, there are some limitations to consider. The study focused on specific crops and diseases, and the generalizability of the methods to other contexts remains to be explored. Additionally, the comparison was based on FID scores, and further evaluation using other metrics and human assessments could provide a more comprehensive understanding of the quality of the generated images. Future research could explore the application of InstaGAN and RePaint to other crops and diseases, assessing their performance across a broader range of scenarios. Additionally, the integration of these methods with existing disease detection models could be investigated to evaluate their impact on detection accuracy. Further refinement of the diffusion process in RePaint and exploration of other generative techniques may also lead to continued improvements in image synthesis quality.

## 11 Conclusion

This study contributes valuable insights into the state-of-the-art methods in the field of generative models for plant disease image synthesis. The introduction of InstaGAN and RePaint, along with their comprehensive evaluation, highlights the potential of these methods as powerful tools for various applications in plant science and computer vision. The findings pave the way for further research and development in this exciting and rapidly evolving field. Through rigorous comparison with state-of-the-art methods on both Tomato and Grape Leaf diseases, the study demonstrated the superior performance of RePaint in generating realistic and high-quality images.

The implications of this work are far-reaching, offering new avenues for data augmentation and the development of more robust disease detection models in plant science. The success of RePaint, in particular, underscores the potential of diffusion-based models in the field of generative models, opening new possibilities for research and application.

Despite the promising results, the study also acknowledged limitations, such as the focus on specific crops and diseases and the reliance on FID scores for evaluation. These areas provide opportunities for future research, including the exploration of other crops, diseases, and evaluation metrics, as well as the integration of these methods with existing disease detection models.

In conclusion, this study marks a significant advancement in the field of generative models for plant disease image synthesis. The introduction and evaluation of InstaGAN and RePaint not only contribute valuable insights into current methodologies but also pave the way for continued innovation and exploration. The findings of this research have the potential to impact various applications in plant science and computer vision, underscoring the importance of continued investment and exploration in this exciting field.

## Data availability statement

Publicly available datasets were analyzed in this study. This data can be found here: https://www.kaggle.com/datasets/mohitsingh1804/plantvillage.

## Author contributions

AM: Conceptualization, Data curation, Formal Analysis, Investigation, Methodology, Project administration, Resources, Software, Validation, Visualization, Writing – original draft. DH: Funding acquisition, Supervision, Validation, Writing – review & editing. ZS: Data curation, Investigation, Validation, Writing – review & editing. KL: Formal Analysis, Investigation, Methodology, Software, Validation, Writing – review & editing.
