# Supplementary material for: Harnessing the power of diffusion models for plant disease image augmentation
Source: Front Plant Sci. 2023 Nov 7;14:1280496. doi: 10.3389/fpls.2023.1280496 (PMC10669158; doi:10.3389/fpls.2023.1280496)
Supplement: Supplementary file 1 [file DataSheet_1.pdf]

# Harnessing the power of diffusion models for plant disease image augmentation

Abdullah Muhammad , Zafar Salman , Kiseong Lee ,and Dongil Han *

Front. Plant Sci., 07 November 2023
Sec. Technical Advances in Plant Science
Volume 14 - 2023 — https://doi.org/10.3389/fpls.2023.1280496

## 1 SUPPLEMENTARY DATA

### 1.1 Tomato Leaf Results

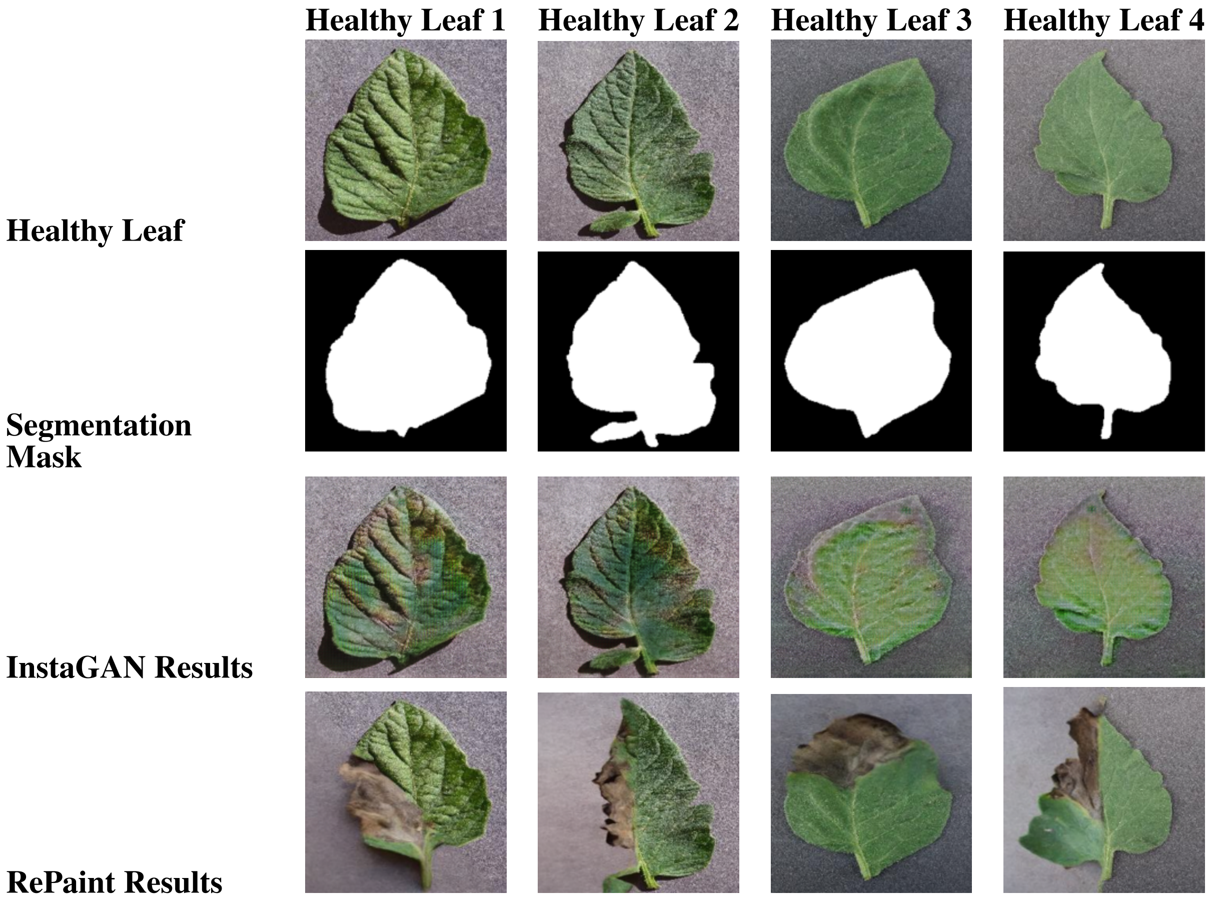


**Figure S1.** This figure shows superior results by RePaint in comparison to InstaGAN in the generation of early blight disease symptoms in healthy tomato leaves. The quality of the image and the definition of disease symptom details are enhanced, successfully synthesizing cut and broken leaves, unlike InstaGAN, which can only copy the style of the target disease but can't change the form and structure to reflect the disease's side effects.

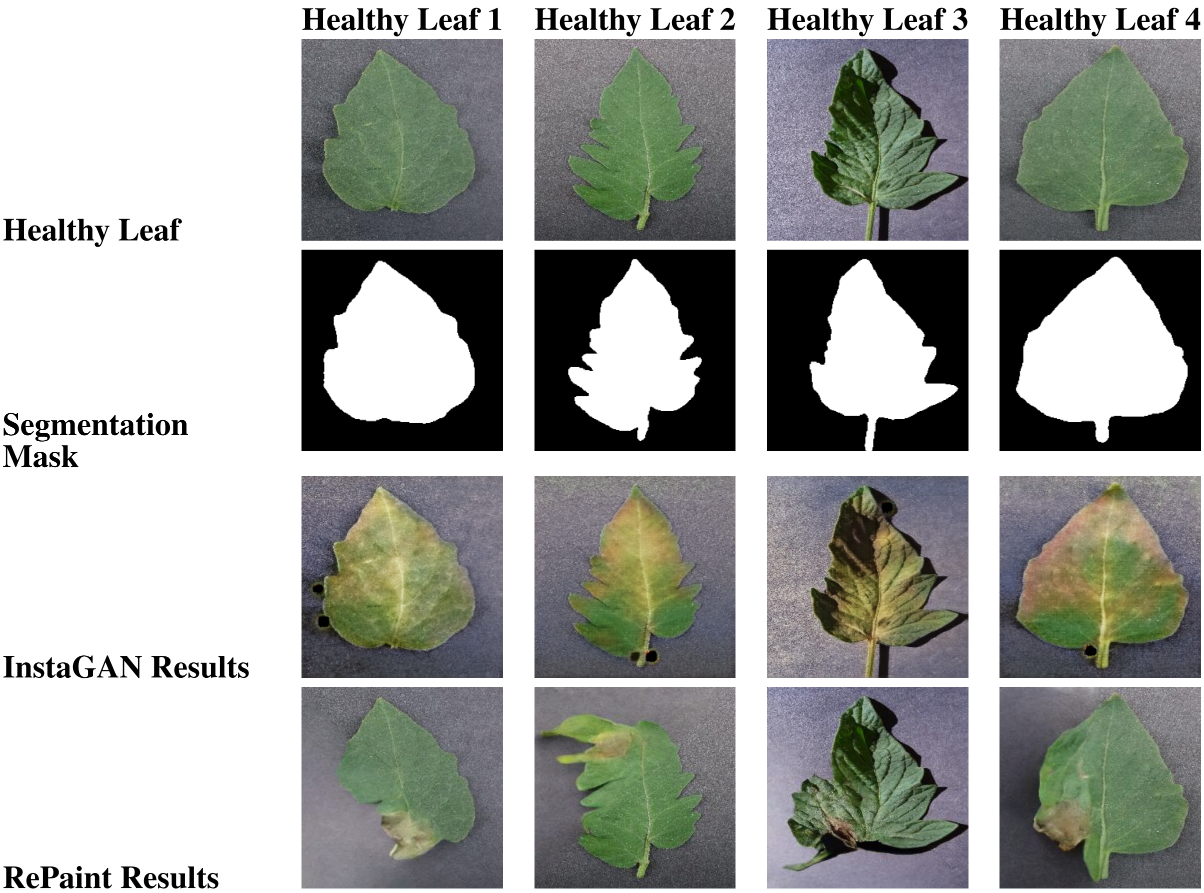


**Figure S2.** This figure presents the challenges and successes of RePaint and InstaGAN in recreating late blight symptoms on healthy leaves. RePaint excels in capturing the intricate patterns and authentic appearance of late blight, overcoming the challenge of its detailed complexity. In contrast, while InstaGAN manages to simulate the disease's presence, it may struggle to depict the fine details that make late blight unique.

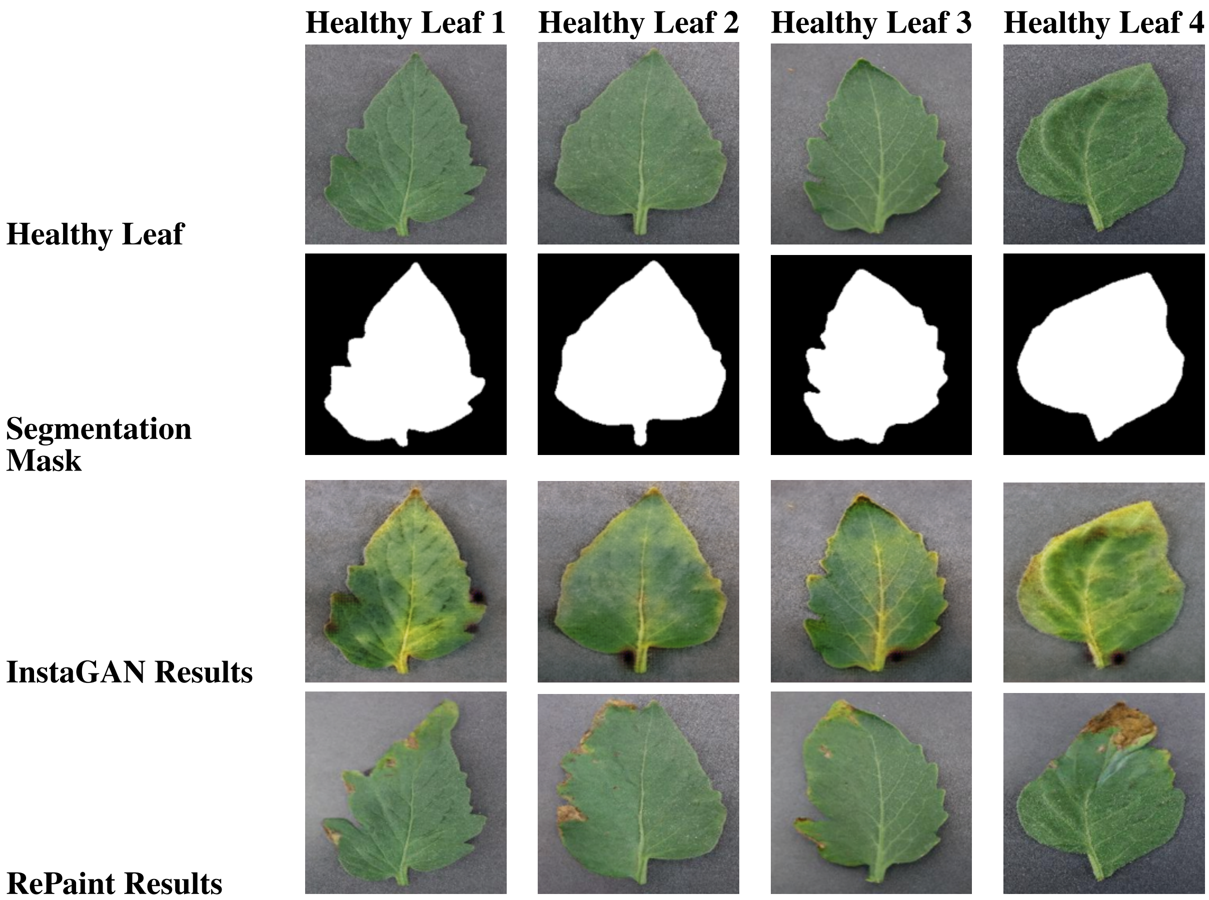


**Figure S3.** This figure illustrates the remarkable performance of RePaint in generating bacterial spot symptoms on healthy tomato leaves. The generated images by RePaint exhibit detailed and realistic disease patterns, capturing the unique characteristics of bacterial spot, while InstaGAN's results lack the intricate details and the natural appearance of the disease.

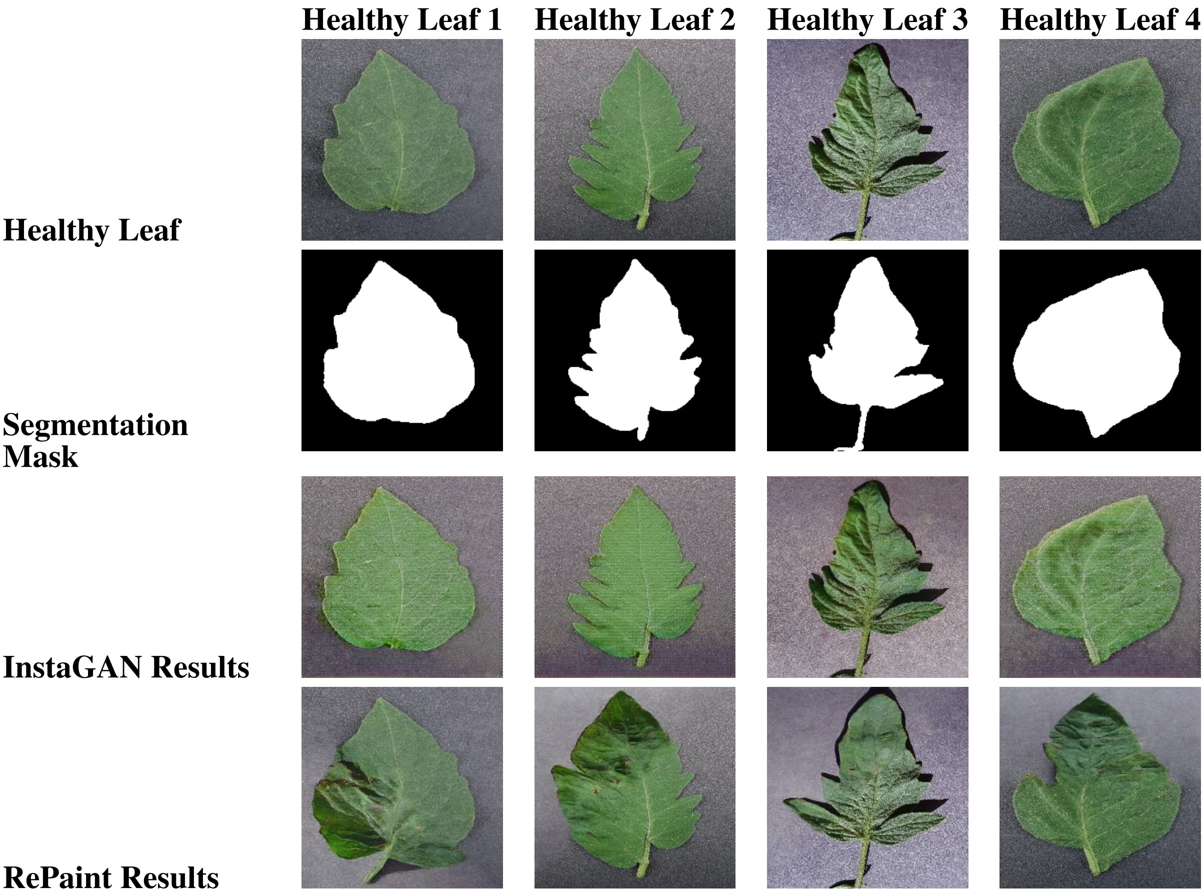


**Figure S4.** This figure highlights the complexities of target spot symptoms and how RePaint and InstaGAN approach these challenges. RePaint's results showcase the characteristic concentric rings and discolorations associated with target spot, providing a highly realistic portrayal of the disease's intricacies. InstaGAN's outcomes may display some ring-like patterns but may not capture the full complexity and nuance of the disease's appearance.

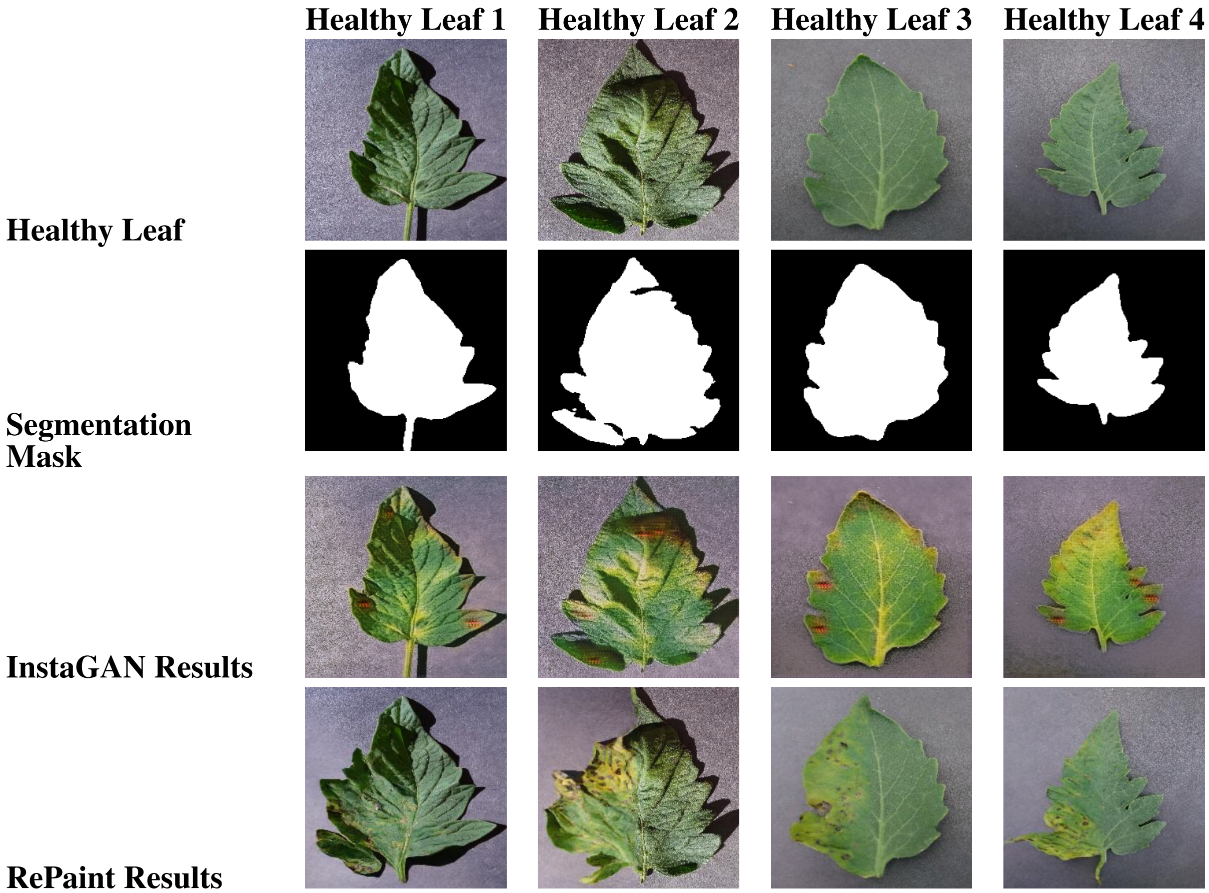


**Figure S5.** This figure delves into the distinctive characteristics of septoria leaf spot and how RePaint and InstaGAN tackle its challenges. RePaint excels in replicating the precise spot patterns and discolorations associated with septoria leaf spot, providing an authentic portrayal of this complex disease. While InstaGAN may exhibit some spot-like effects, it may struggle to capture the full intricacy of the symptoms.

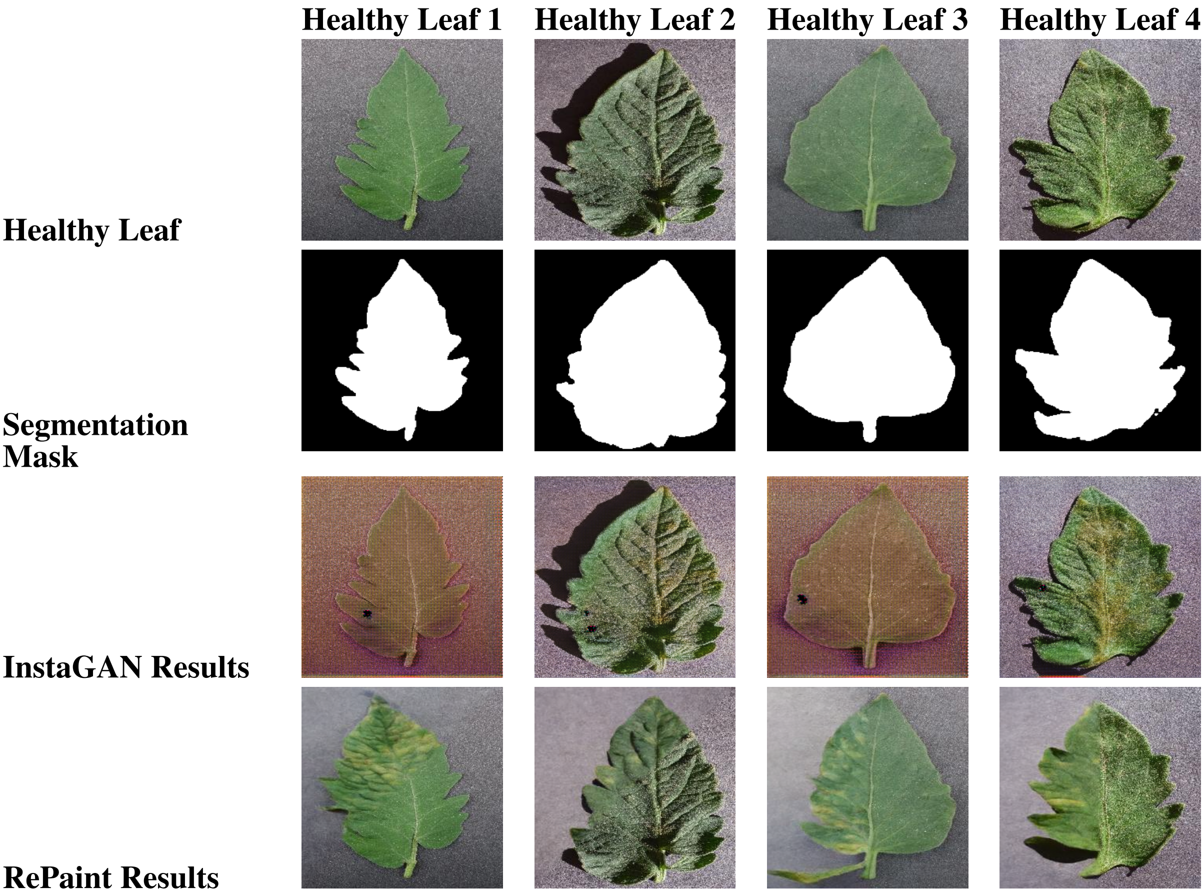


**Figure S6.** In this exploration of spider mites two-spotted symptoms, we observe how RePaint and InstaGAN address the challenges posed by this disease. RePaint excels in replicating the distinctive patterns and discolorations caused by spider mites, offering a convincing representation of the disease's complexity. While InstaGAN may capture some aspects of the disease, it may not fully convey the intricate details that define spider mites two-spotted.

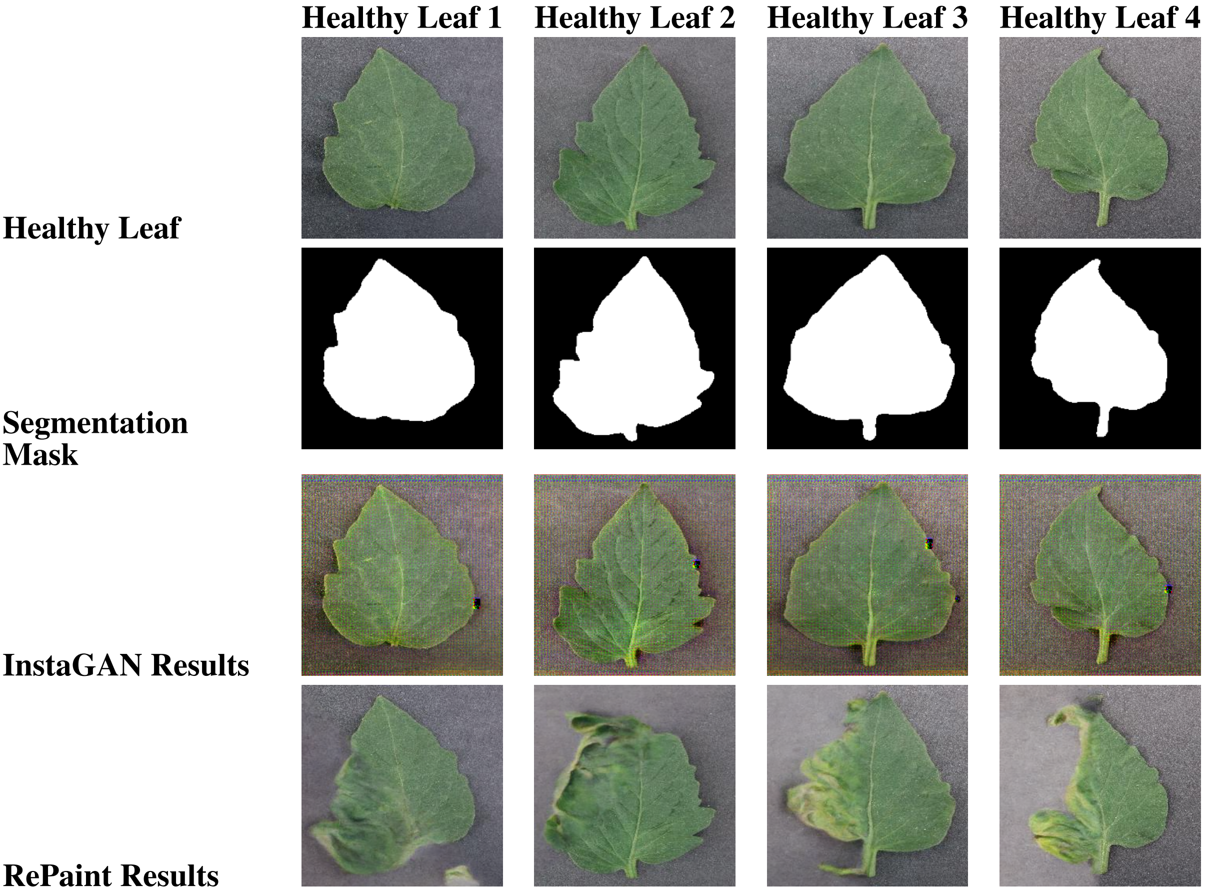


**Figure S7.** This figure delves into the challenges posed by yellow leaf curl virus symptoms and how RePaint and InstaGAN address them. RePaint's results capture the characteristic curling and yellowing of leaves, offering a highly authentic portrayal of the disease's complexities. While InstaGAN may simulate some aspects of the disease, it may struggle to convey the full complexity and nuances of yellow leaf curl virus symptoms.

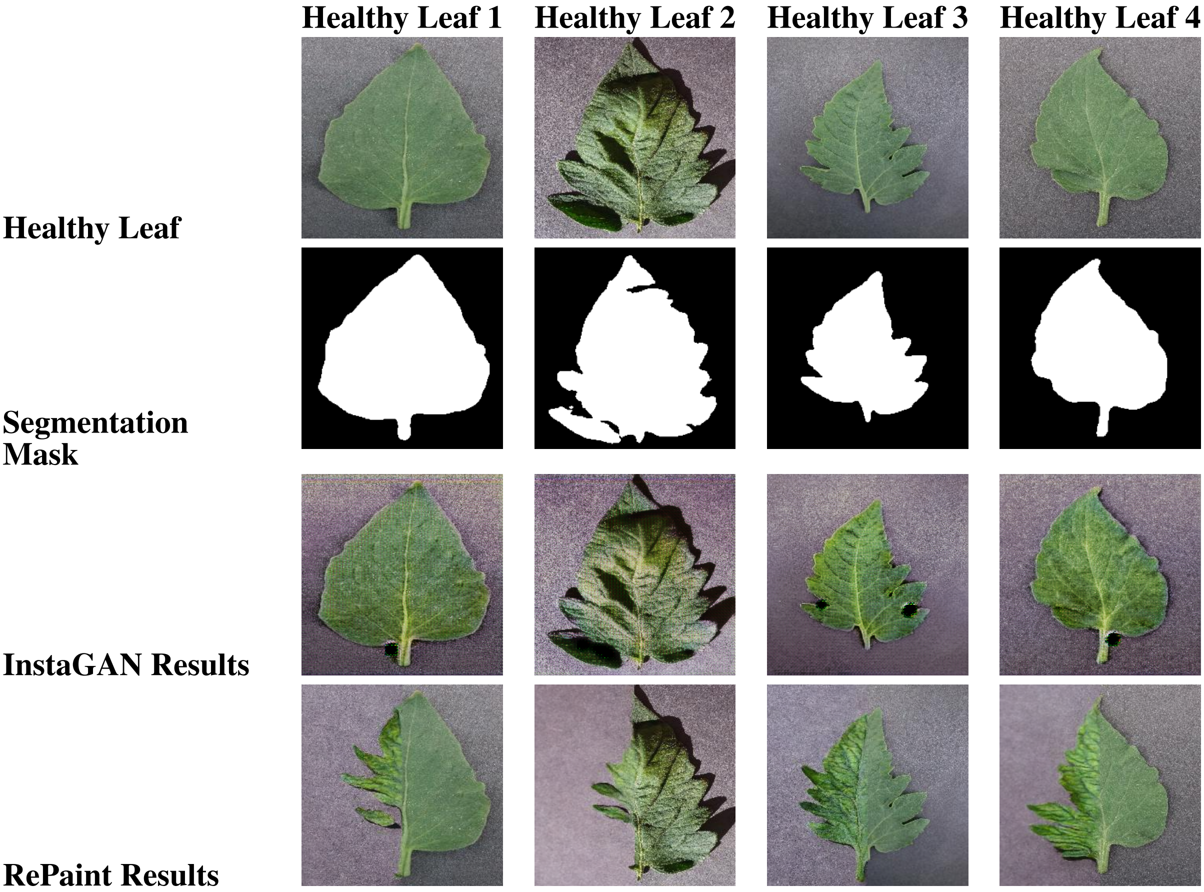


**Figure S8.** In this figure, we explore the complexities of mosaic virus symptoms and the capabilities of RePaint and InstaGAN in recreating them. RePaint's images accurately depict the mosaic patterns and discolorations typical of mosaic virus, delivering a convincing representation of the disease's intricacies. While InstaGAN may simulate some aspects of the disease, it may not fully convey the level of detail and realism achieved by RePaint.

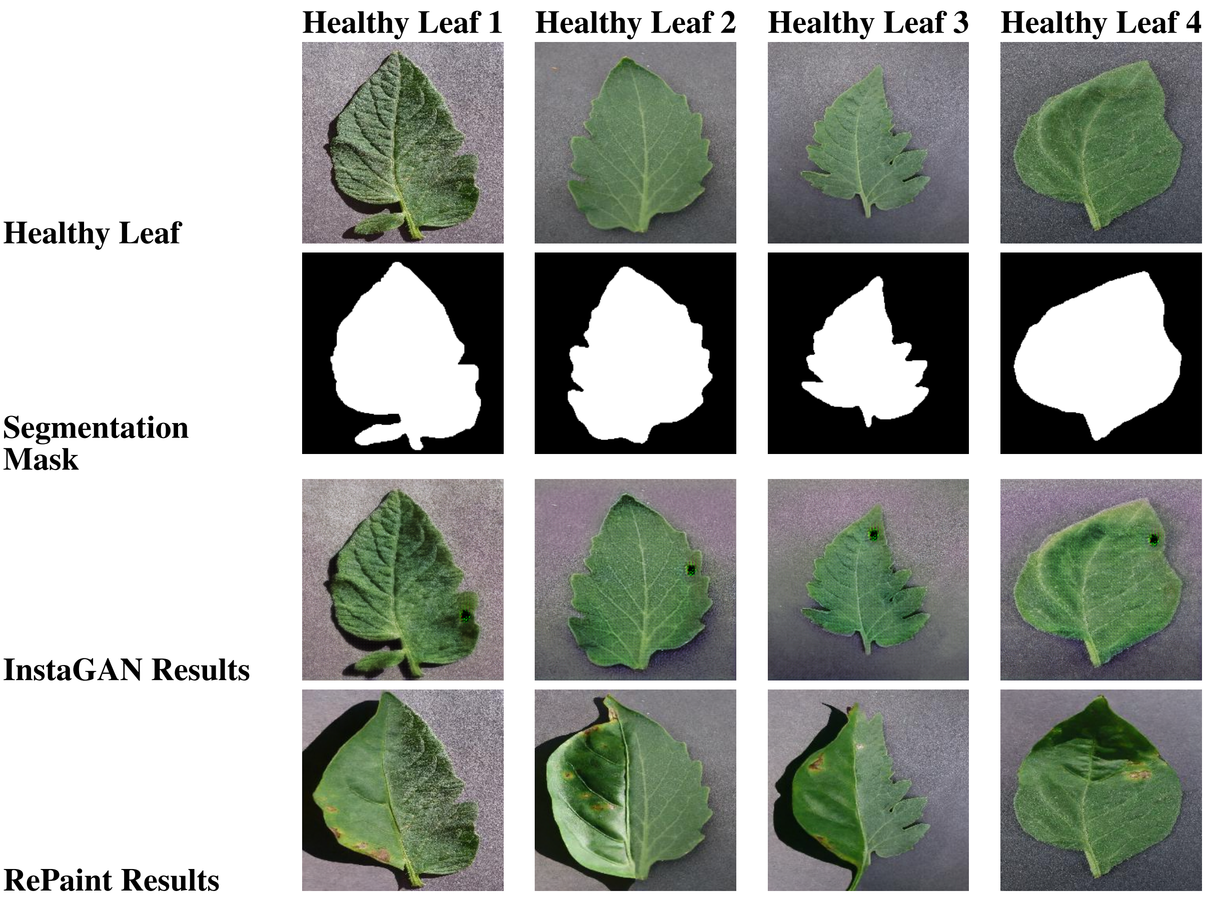


**Figure S9.** In this figure, we explore the intricacies of leaf mold symptoms and the capabilities of RePaint and InstaGAN. RePaint adeptly reproduces the challenging mold patterns, offering a convincing representation of the disease's intricacy. InstaGAN, though attempting to emulate the disease style, may find it challenging to convey the nuanced details that define leaf mold.

## 1.2 Grape Leaf Results

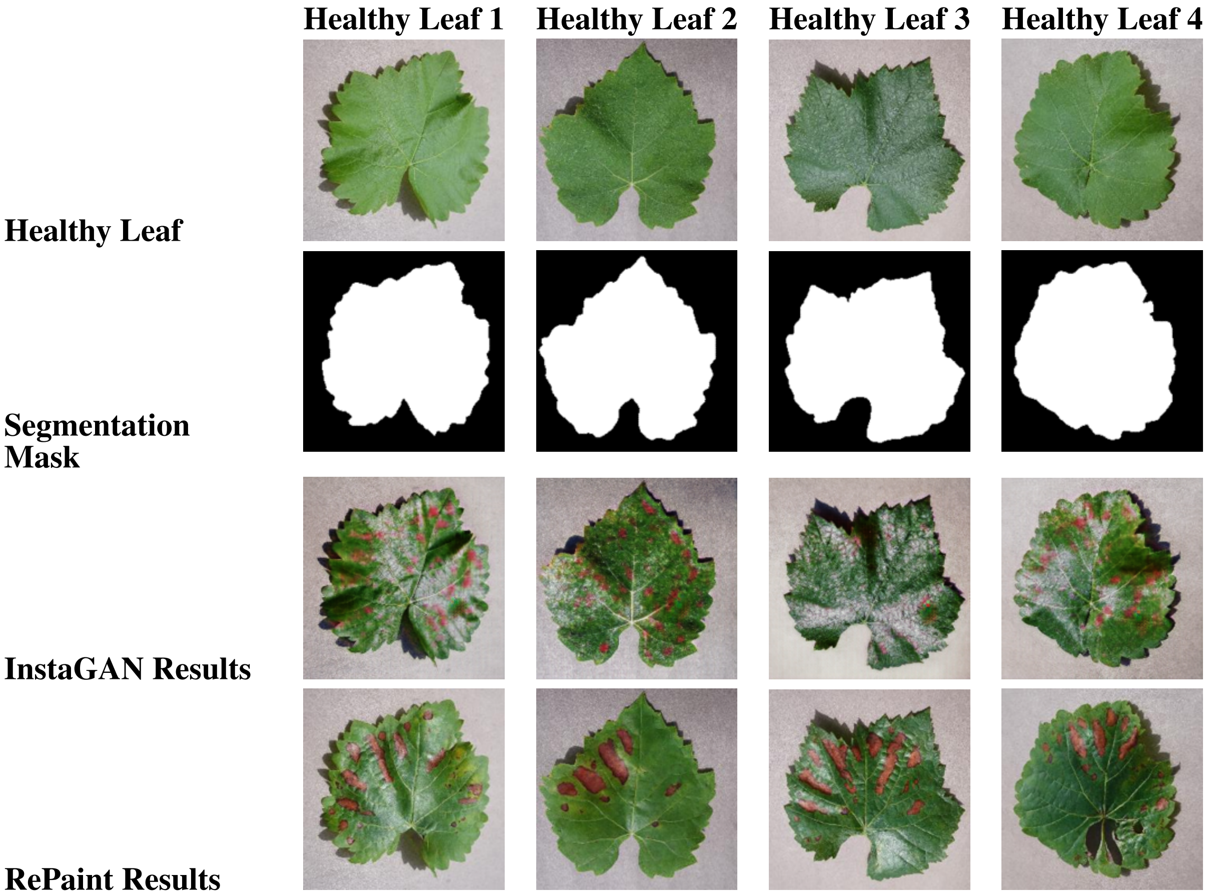


**Figure S10.** This figure highlights the superiority of RePaint in simulating black measles symptoms on healthy grape leaves. RePaint's generated images showcase the distinct features of black measles, including the characteristic dark spots and deformations, outperforming InstaGAN, which struggles to replicate the true nature and severity of the disease.

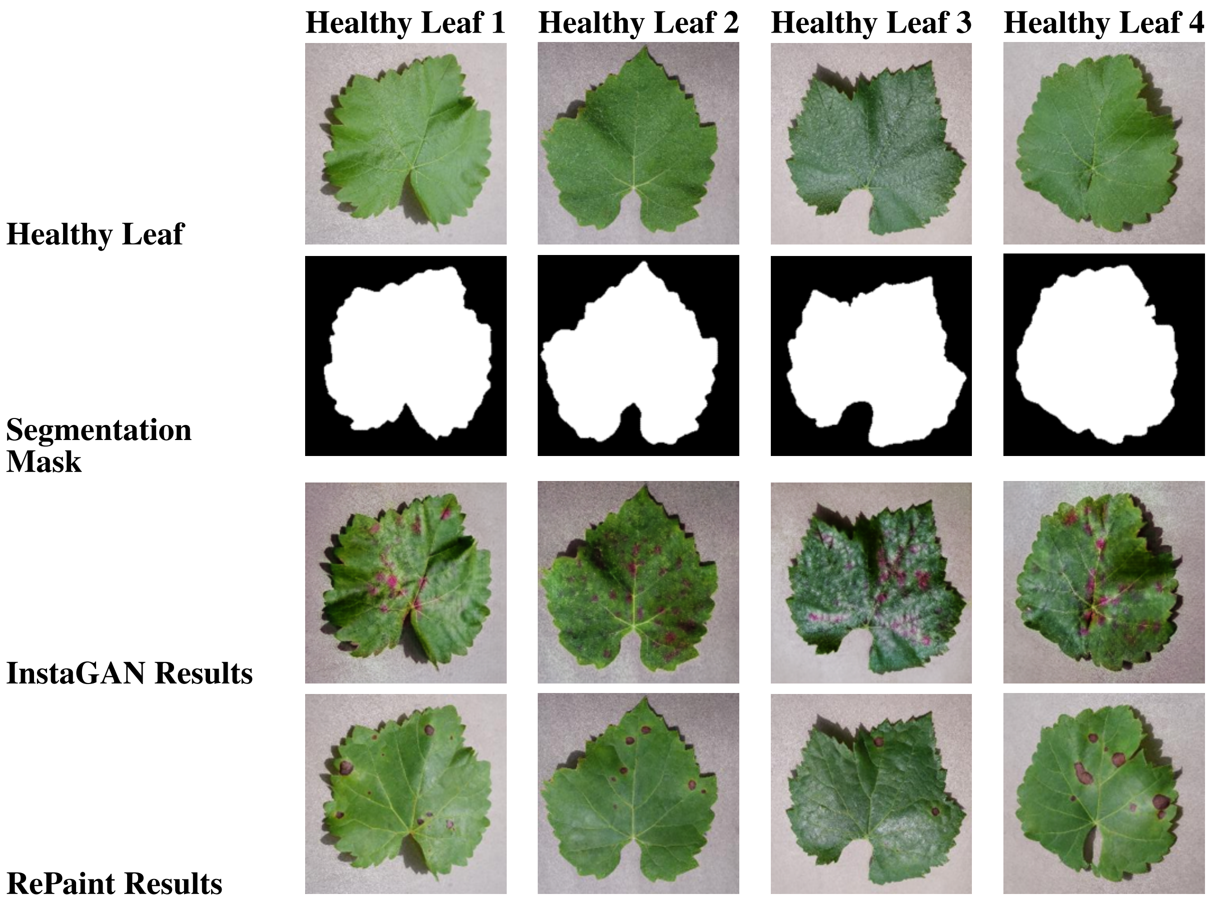


**Figure S11.** This figure demonstrates RePaint's ability to accurately generate black rot symptoms on healthy grape leaves. The images produced by RePaint are rich in texture and closely resemble the actual appearance of black rot, whereas InstaGAN's results fall short in capturing the complexity and authenticity of this particular disease.

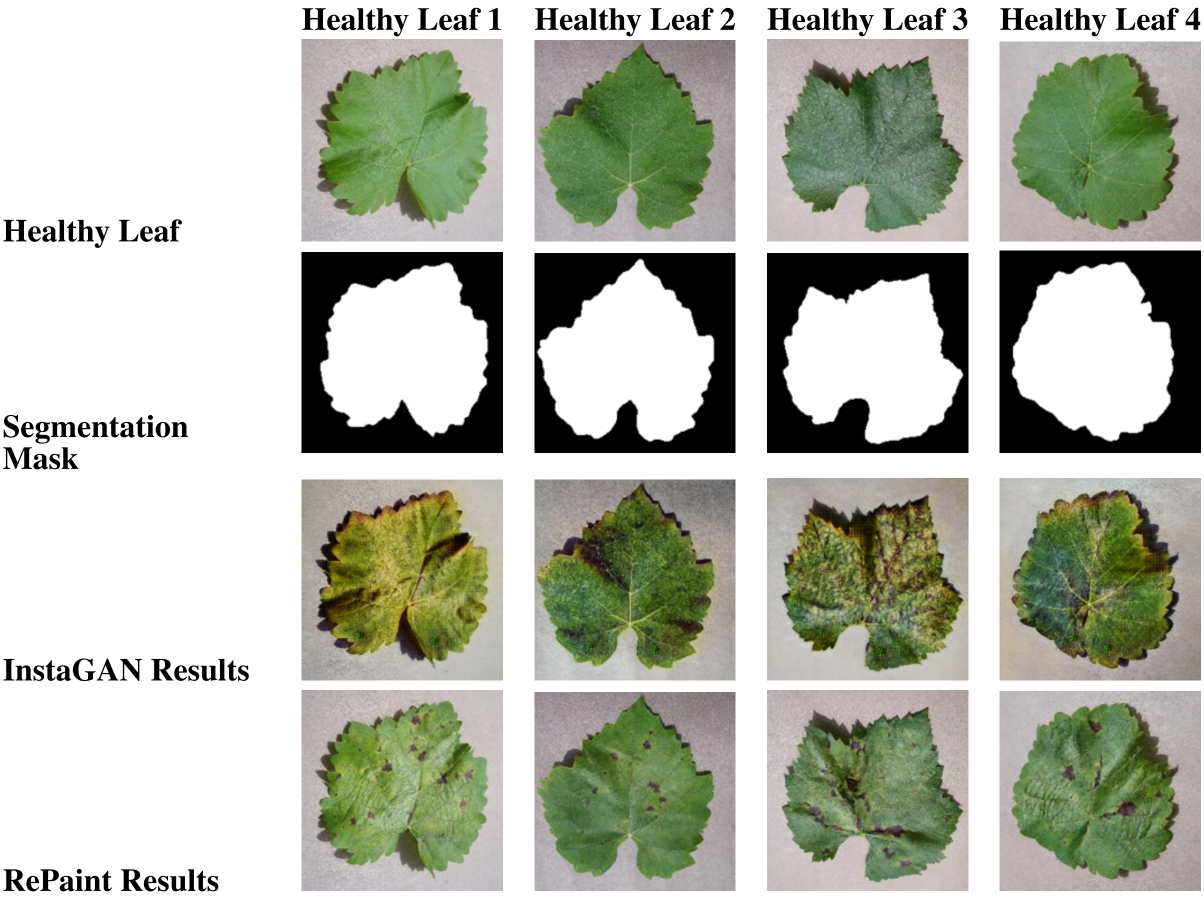


**Figure S12.** This figure presents the impressive capability of RePaint in creating leaf blight symptoms on healthy grape leaves. RePaint's results are characterized by the precise reproduction of the blight's discoloration and decay, offering a more convincing representation of the disease compared to InstaGAN, which fails to capture the full extent of the leaf blight's impact.
